# Variant Plateau’s law in atomically thin transition metal dichalcogenide dome networks

**DOI:** 10.1038/s41467-023-36565-2

**Published:** 2023-02-24

**Authors:** Boqing Liu, Tanju Yildirim, Tieyu Lü, Elena Blundo, Li Wang, Lixue Jiang, Hongshuai Zou, Lijun Zhang, Huijun Zhao, Zongyou Yin, Fangbao Tian, Antonio Polimeni, Yuerui Lu

**Affiliations:** 1 School of Engineering, College of Engineering and Computer Science, The Australian National University, Canberra, ACT 2601 Australia; 2 Center for Functional Sensor & Actuator (CFSN), Research Center for Functional Materials, National Institute for Materials Science (NIMS), 1-1 Namiki, Tsukuba, Ibaraki 305-0044 Japan; 3 Department of Physics and Institute of Theoretical Physics and Astrophysics, Xiamen University, Xiamen, 361005 China; 4 Dipartimento di Fisica Sapienza Università di Roma, 00185 Roma, Italy; 5 School of Engineering and Information Technology, University of New South Wales, Canberra, ACT 2600 Australia; 6 Centre for Catalysis and Clean Energy, Gold Coast Campus, Griffith University, Queensland, 4222 Australia; 7 State Key Laboratory of Integrated Optoelectronics, School of Materials Science and Engineering, and Jilin Provincial International Cooperation Key Laboratory of High-Efficiency Clean Energy Materials, Jilin University, Changchun, 130012 China; 8 Research School of Chemistry, College of Science, The Australian National University, Canberra, ACT 2601 Australia; 9 ARC Centre of Excellence in Quantum Computation and Communication Technology ANU node, Canberra, ACT 2601 Australia

**Keywords:** Two-dimensional materials, Two-dimensional materials

## Abstract

Since its fundamental inception from soap bubbles, Plateau’s law has sparked extensive research in equilibrated states. However, most studies primarily relied on liquids, foams or cellular structures, whereas its applicability has yet to be explored in nano-scale solid films. Here, we observed a variant Plateau’s law in networks of atomically thin domes made of solid two-dimensional (2D) transition metal dichalcogenides (TMDs). Discrete layer-dependent van der Waals (vdWs) interaction energies were experimentally and theoretically obtained for domes protruding in different TMD layers. Significant surface tension differences from layer-dependent vdWs interaction energies manifest in a variant of this fundamental law. The equivalent surface tension ranges from 2.4 to 3.6 N/m, around two orders of magnitude greater than conventional liquid films, enabling domes to sustain high gas pressure and exist in a fundamentally variant nature for several years. Our findings pave the way towards exploring variant discretised states with applications in opto-electro-mechanical devices.

## Abstract (web summary)

Plateau’s law describes the configuration (joint angles ~120°) of soap bubbles, foams and cellular structures, but its applicability to solid films has not been explored. Here, the authors report the observation of a variant Plateau’s law in networks of nanobubbles made of 2D semiconductors, measuring largely varying joint angles due to the thickness-dependent effective surface tension.

## Introduction

Since Joseph Plateau’s fundamental observations^1^ of soap bubbles in the 19th century revealed their borders meet at equal joint angles of 120°, this law has been a prominent physical law in natural science. Apart from soap bubbles^1–6^, Plateau’s law naturally occurs in liquid foams such as beer froth, and in solid foams such as cells, honeycombs and metallic foams^7–10^. Even in emerging two-dimensional (2D) materials such as graphene^11^, the atomic arrangement of carbon atoms form a hexagonal structure at 120° to assume an equilibrated state^12^. Whilst some studies have shown that the joint angles in bubbles and foams are not always 120°, the angle deviation is quite small of only a few degrees^13–16^. Due to the low surface tension (*σ*) of liquids, such as 0.07–0.09 N/m for water^17^ and 0.025 N/m for soap bubbles^18^, the capped pressure in liquid bubbles is low and they only exist for short periods of time before bursting.

On the other hand, atomically thin layered materials such as graphene^11^ and transitional metal dichalcogenides (TMDs)^19,20^ are becoming promising candidate materials for the creation of nano-bubbles^21–26^. These 2D mono- and few-layer^27,28^ materials are at least two orders of magnitude thinner than soap bubble films^29^, but have shown strong mechanical strength^30,31^, high resistance to common alkaline and acids^32,33^, as well as fabulous chemical and thermal stability^34^. All previously demonstrated nano-bubbles in 2D materials were isolated singular ones without networking topologies^21–25^.

Herein, we successfully created atomically thin TMD dome networks and observed variant Plateau’s law in nano-scale solid systems. Variant Plateau’s law is due to thickness dependent interlayer adhesion energy between the first few interacting TMD layers and the basal plane, causing thickness dependent stiffness values depending on dome layer number, resulting in large effective surface tension differences, which leads to the formation of large joint angle differences of approximately 77° between the largest and smallest angles. Results contrast with the commonly observed equal joint angles and 0° angle difference commonly associated with Plateau’s law, demonstrating that this variant is observed due to our 2D TMD dome networks.

## Results and discussion

### Generation and characterisations of TMD pressurised domes

**Fig. 1:**
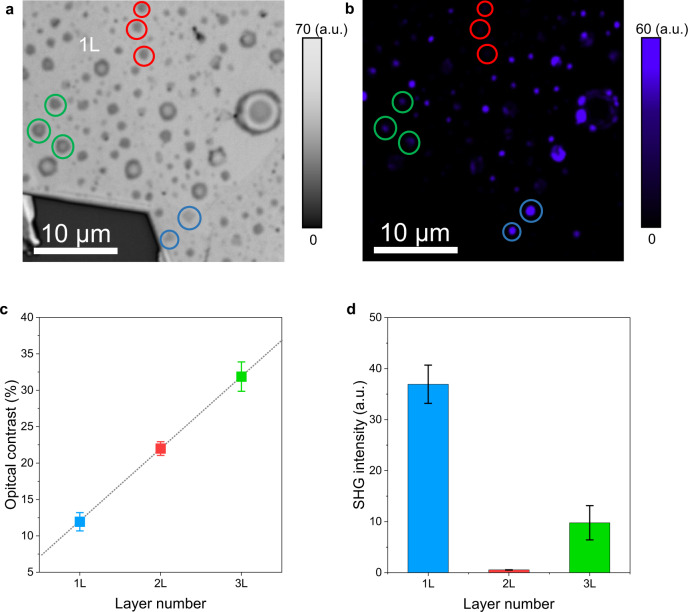
Optical characterisation of mono- and few-layer pressurised nano-domes in TMDs. **a**, Optical microscope image of pressurised domes generated on a WS_2_ flake, whereby mono- (1 L), bi- (2 L) and tri-layer (3 L) domes have been highlighted by blue, red and green circles, respectively. The layer number of the dome is initially identified from the optical contrast. **b** Second harmonic generation (SHG) mapping of the same flake shown in **a**. 1 L domes show the strongest SHG signal, whilst 2 L domes are almost invisible under the same excitation and collection conditions. **c** Measured optical contrast as a function of dome layer number. The measured optical contrast for 1–3 L domes with similar sizes are 11.9% ± 1.3% (blue), 22.0% ± 0.9% (red) and 31.9% ± 2.0% (green), respectively. The error bars represent statistical variation from at least 4 domes for each group with different layer number. **d** Measured SHG intensity as a function of layer number of the domes. The measured SHG intensities for 1–3 L domes with similar sizes are 36.93 ± 3.74, 0.54 ± 0.01 and 9.79 ± 3.36 arbitrary unit (a.u.), represented by blue, red and green bars, respectively. The error bars represent statistical variation from at least 5 domes from each group with different layer number.

Recently, we successfully used a low-energy (<15 eV) proton irradiation technique to produce pressurised and spherical monolayer hydrogen domes on the surface of bulk TMDs^25^. Here, we increased the proton beam energy to ~25 eV, which allowed protons to deliberately penetrate deeper basal planes of the TMD, leading to the creation of mono-(1 L), bi-(2 L), and tri-layer (3 L) pressurised hydrogen nano-domes (Fig. 1). Dome layer number was initially identified from optical contrast (Fig. 1a) and further confirmed by second harmonic generation (SHG) imaging performed on the same flake (Fig. 1b). The optical contrast showed a linear relationship with layer number (Fig. 1c), consistent with a previous report^35^. 1 L and 3 L domes exhibit intense and less intense SHG signals, respectively, and 2 L domes show no SHG (Fig. 1d), which is attributed to the fact that for 2H phase TMDs, broken inversion symmetry only exits in samples with odd layer number^36,37^. The percentage yield of 2 L and 3 L domes was enhanced under higher proton dosage, obtained via statistical analysis (Supplementary Fig. 1).

**Fig. 2:**
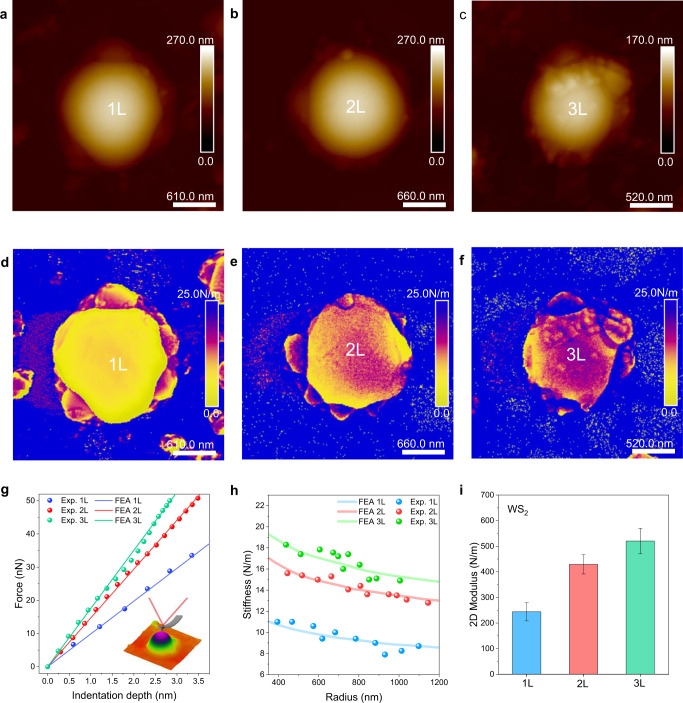
Mechanical characterisations of mono- and few-layer pressurised TMD nano-domes. **a**–**c** Atomic force microscope (AFM) images of a 1 L (**a**), 2 L (**b**) and 3 L dome (**c**). The measured centre dome height (*h*_*m*_) to radius (*R*) ratio (*h*_*m*_*/R*) for 1–3 L domes are 0.18, 0.17, and 0.16, respectively. **d**–**f** Stiffness mapping images measured for the 1–3 L domes shown in **a**–**c**. **g** Force-indentation curves (dots) measured at the centre of domes with different layer numbers (1–3 L). The simulated force-indentation curves (solid lines) from finite element analysis (FEA) match very well with the measured ones. The inset shows the experimental set up for data acquisition: an AFM tip was used for the mechanical nano-indentation of domes. **h** Measured stiffness as a function of dome radius (solid dots), for 1–3 L domes. Simulated results (lines) by FEA match well with experimental observations. **i** Extracted two-dimensional modulus (*E*_2D_) of WS_2_ as a function of layer number. The extracted *E*_2D_ values are 244.0 ± 35.7, 429.5 ± 37.7 and 520.4 ± 49.2 N/m for 1–3 L WS_2_ domes, respectively. The error bars represent statistical variation from 20 domes for each group with different layer number.

Due to the resolution limit of conventional optical microscopes, distinguishing the layer number of small-sized domes is challenging. Therefore, AFM height imaging (Fig. 2a–c) and stiffness mapping images of the domes were obtained and used to depict the layer dependent mechanical properties (Fig. 2d–f). Given the sample domes’ similar radii (500–630 nm), the obtained stiffness images reveal a substantial relationship between layer number and mechanical properties. An inferred method based on a strong boundary condition (detailed in Supplementary Note 1)^38,39^ was used to calculate the 2D modulus (*E*_2D_) of the domes formed in different layers. Finite element analysis (FEA) (Supplementary Note 2) was compared against the experimental data for the nano-indentation process using the derived *E*_2D_, illustrating good agreement (Fig. 2g). The measured stiffness showed a maximum value at the dome centre and decreased gradually by 20–30% as the AFM tip moved away from centre to the edge, which also matched with simulation results from FEA and analytically (Supplementary Fig. 2). During indentation, there should be a resultant backward pressure applied due to the internal pressure encapsulated by a dome, and this may explain slight discrepancies^38,39^; however, overall, this does not appear to be a major influence as the FEA accounts for both pressures and a good agreement is found at the centre of domes. Slight discrepancies arise when moving away from the dome centre, which may be due to the non-uniform pressure distribution acting on the nano-indenter due to dome curvature. Moreover, the measured stiffness (at the dome centre) of different 1 L, 2 L and 3 L domes exhibit an exponentially decaying trend with increasing dome radius for a constant *E*_2D_, which agrees well with the FEA simulation as shown in Fig. 2h. FEA was also conducted for slightly modified dome footprint shapes which showed comparable stiffness values with a perfect circular dome footprint (Supplementary Fig. 3 and Supplementary Note 3). The gas pressure of domes also possess an exponential trend, leading to higher pressure up to tens of MPa capped in smaller and stiffer domes (see Supplementary Fig. 4). Moreover, we could estimate the pressure of domes at low *T* to verify the inferred method against the phase transition of H_2_ molecules, showing good agreement as in Supplementary Fig. 5 and Supplementary Note 4.

Thus, using layer-dependent stiffness curves and dome radius, we can quickly identify thickness and mechanical properties of domes that match well with FEA (Fig. 2g, h). Figure 2i reveals that the 2D modulus monolithically increases with layer number for 1–3 L WS_2_ domes, consistent with the trend reported for suspended membranes^30^. Similarly, we also used the inferred indentation method to experimentally obtain the 2D modulus of MoS_2_ domes shown in Supplementary Fig. 6a.

### Resolving layer-dependent adhesion energies in TMDs by domes

**Fig. 3:**
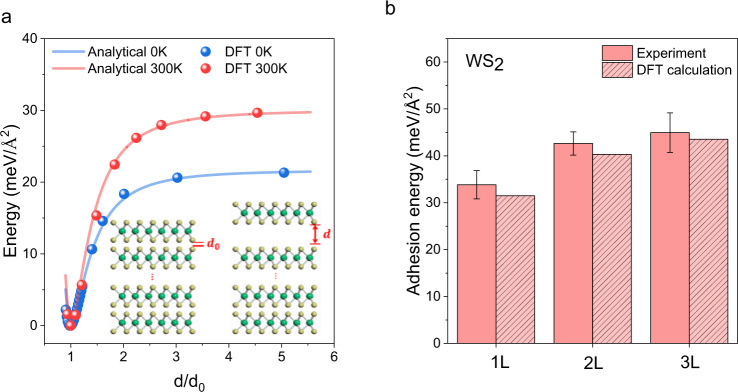
Resolving layer-dependent adhesion energies in TMDs by nano-domes. **a** Calculated energy required to separate a WS_2_ monolayer from the bulk as a function of separation distance (*d*), by DFT (dots) and analytical solution (line), at 0 (blue) and 300 K (red). *d*_*0*_ is the initial separation distance. The adhesion energy is defined to be the energy per area required to separate the layer from the bulk. The adhesion energies of a WS_2_ monolayer at 0 and 300 K are calculated to be 21.5 and 29.7 meV/Å^2^, respectively. The analytical solution has the form ${{{{{{\rm{\gamma }}}}}}}_{{dist}}=\,{{{{{\rm{\gamma }}}}}}\left[\frac{3}{2}{(\frac{{d}_{0}}{d})}^{3}-\frac{1}{2}{(\frac{{d}_{0}}{d})}^{9}\right]$, where *γ*_*dist*_ is the monolayer-surface interaction energy per unit area at a corresponding separation distance and *γ* is the interfacial adhesion energy per unit area^45^; The inset provides the schematic illustration of the initial interlayer distance (*d*_*0*_) and modified separation distance (*d*) in WS_2_. **b** Measured layer-dependent adhesion energies of WS_2_, which are the energies required to exfoliate top layers (1–3 L) from a WS_2_ bulk flake, using nano-domes. The adhesion energy values obtained from 1–3 L WS_2_ are 33.8 ± 3.0, 42.6 ± 2.5 and 45.0 ± 4.2 meV/Å^2^, all of which are in good agreement with the DFT results of 31.5, 40.3 and 43.5 meV/Å^2^, respectively. The error bars represent statistical variation from 20 domes for each group with different layer number.

The vdW adhesion energy (*γ*) plays an important role in layered materials and their physical properties, and several techniques have been reported to measure the adhesion energy between monolayer materials and the substrate^40–44^, *γ* in this work refers to the interlayer adhesion between TMD basal planes from the remaining bulk flake and not the base substrate. The adhesion energies in layered materials should be layer dependent, particularly for mono- and few-layer domain structures, but this layer-dependence was not previously explored. Here, our 1 L, 2 L, and 3 L domes provide a fascinating platform to probe the intrinsic and discrete layer-dependent adhesion energies in TMDs. We firstly used density functional theory (DFT) (Supplementary Note 5) to calculate the *γ* values for 1 L WS_2_ at 0 and 300 K (Fig. 3a). The calculated *γ* value at 0 K is consistent with previously reported values^45^; the calculated *γ* value at 300 K is ~50% higher than that at 0 K, consistent with recent results from experiments that *γ* is enhanced at higher temperature^40^. The *γ* values for 1 L, 2 L and 3 L WS_2_ were experimentally determined based on the material properties of the nano-domes (Supplementary Note 1), which showed a clear layer-dependence, matching well with our DFT calculated values at 300 K, including the presence of trapped hydrogen molecules (Fig. 3b). Minor differences may be attributed to two reasons; the first being the variation in the bond length of the topmost layers and inter-layer spacing induced by the external pressure, and thermal effects in ambient conditions^46^. Another reason may be modulated mechanical properties caused by trapped hydrogen molecules^47^. DFT results revealed that adsorbed hydrogen molecules affect the 2D modulus of monolayer TMDs, but have a small effect in few layer TMDs (Supplementary Fig. 7). This explains the larger discrepancy between experimental values of adhesion energy and DFT calculated values in monolayer domes, whereas few layer domes have negligible difference. Similarly, the experimental *γ* values for 1 L, 2 L and 3 L MoS_2_ are discrete and layer-dependent (Supplementary Fig. 6b). Our *γ* values for TMD monolayers (Fig. 3b and Supplementary Fig. 6b) are consistent with previously reported experimental results^40^.

### Identification of joint bi-dome structure and configurations

**Fig. 4:**
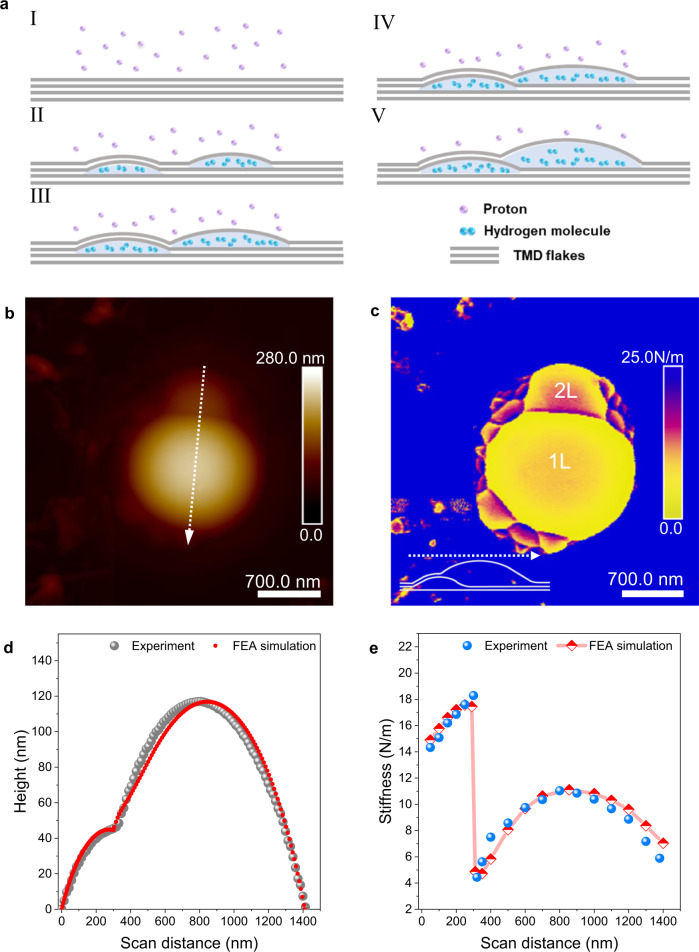
Identification of structure configuration of a joint bi-dome via nano-indention. **a** Schematic illustration of a joint bi-dome formation process. **b** AFM image of a WS_2_ joint bi-dome made of a large 1 L dome and small 2 L dome. **c** Stiffness mapping of the joint bi-dome shown in **a**. The inset indicates the structure of the domes along the white dashed line in **b**. **d** Measured height profile (grey) of the joint bi-dome along the white dashed line shown in **b**. The simulated profile (red) generated by FEA reasonably matches with the measured one. **e** Measured stiffness (solid blue dots) as a function of scan distance along the white dashed line shown in **b**. Simulated stiffness values by FEA calculation (red diamond) match well with experimental values. The sharp drop of stiffness value at 300 nm corresponds to the joint boundary between 2 L and 1 L domes.

When the exfoliated TMD thick flake is exposed to ~25 eV proton irradiation, hydrogen protons penetrate the top few basal planes of the bulk TMD flake, and then reduce to form hydrogen molecules accumulated in the top few TMD layers, forming mono- and few-layer domes (Fig. 4 a Stage I-II). With longer proton exposure time, the domes grow and interface with each other, and the inner pressure from the 1 L dome (on the right) overcomes the vdWs adhesion force and lifts off the top-most layer partially covering the adjacent 2 L dome (on the left), eventually forming a joint double dome (bi-dome) system (Fig. 4 a Stage III-V). Therefore, the hydrogen molecules in our 1 L, 2 L and 3 L domes exist in different basal planes of the TMD, leading to intriguing different joint dome configurations and interaction dynamics among domes. It is important to note here that the substrate is the TMD flake beneath the resulting domes and not the base SiO_2_/Si substrate, as growing domes on different base substrates still results in a universal height to radius ratio^25^. Experimentally, many different coalescing dome formations were observed, and the most common case was the bi-dome presented in the AFM image as shown in Fig. 4b. This case is the existence of a bi-dome composed of a large 1 L dome coinciding with a smaller 2 L dome, demonstrating complex interaction between hydrogen accumulation underneath different basal planes of the TMD. To maintain stability, domes sharing common vertices must be in different layers of the TMD; otherwise, domes will merge resulting in larger domes in the same basal plane to minimise their surface energy, analogous to liquid bubbles. The structure and configuration of a joint bi-dome was directly confirmed by a bursting experiment test (Supplementary Fig. 8 and Supplementary Note 6). To further characterise the structure and confirm the configuration, stiffness mapping was conducted among the joint domes and a clear dependency between the stiffness of interacting domes was observed (Fig. 4c). This reaffirms domes are in different layers as illustrated by the inset in Fig. 4c, where the smaller 2 L dome exhibits much higher stiffness than the larger 1 L dome. FEA simulations of the generated height profile and stiffness of the two merging domes (with configuration depicted by the inset in Fig. 4c) is given in Fig. 4d, e, respectively, illustrating good agreement between the simulation and experimental results. Some discrepancies near the transition region are visible, which is attributable to the AFM resolution or a mismatch in modelling the geometry and boundary conditions. This demonstrates that domes function as highly pressurised membranes with high tension across the dome interface between internal and external pressures (Supplementary Note 7). Moreover, a very sharp stiffness transition edge between dome layers is visible (Fig. 4e), further confirming the two domes are sitting in different basal planes. The same method was applied to a different joint configuration with a larger 2 L region and smaller 1 L region, also showing good agreement between simulation and experiment (Supplementary Fig. 9).

### Observation of variant Plateau’s law in joint dome systems

**Fig. 5:**
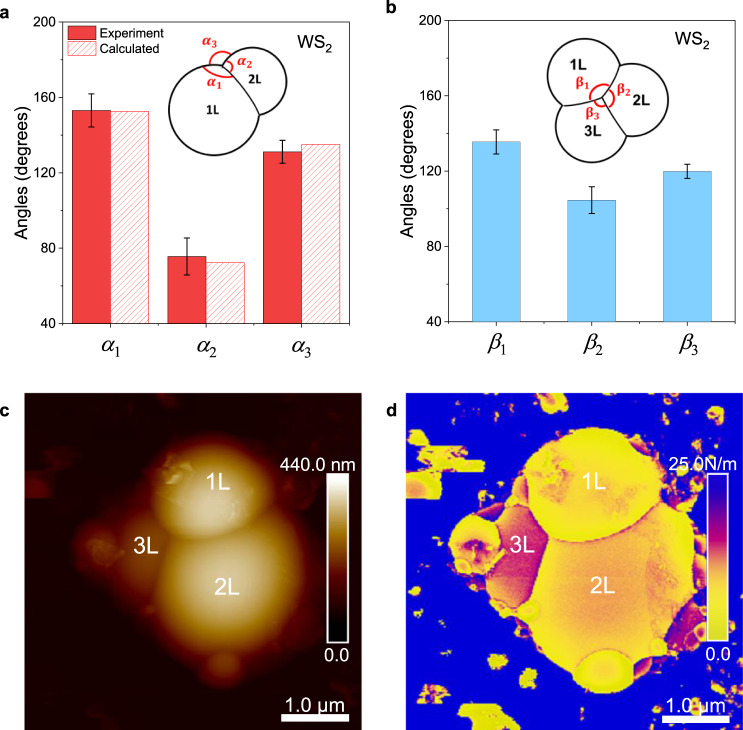
Observation of variant Plateau’s law in bi- and tri-dome systems. **a** Histogram of the joint angles (*α*_*i*_, *i* = 1, 2, 3) in WS_2_ bi-dome systems, extracted experimentally (solid bars) and analytically (patterned bars). The inset shows the angle notations in a standard bi-dome configuration. The experimental values for joint angle are 153.1° ± 8.8°, 75.6° ± 9.8° and 131.2° ± 6.1° for *α*_1_, *α*_2_ and *α*_3,_ respectively. Statistical data was collected from 20 joint bi-domes. The calculated values for *α*_1_, *α*_2_ and *α*_3,_ are 152.6°, 72.3° and 135.1°, respectively, all of which are in good agreement with experimental values. **b** Histogram of the joint angles (*β*_*i*_*, i* = 1, 2, 3) in WS_2_ tri-dome systems, extracted experimentally. The inset shows the angle notations in a standard tri-dome configuration. The experimental values for joint angle are 135.5° ± 6.4°, 104.6° ± 7.1° and 119.9° ± 3.7° for *β*_1_, *β*_2_ and *β*_3_, respectively. Statistical data was collected from 15 joint tri-domes. **c** AFM image of a WS_2_ tri-dome system, consisting of 1 L, 2 L and 3 L domes joint together. **d** Stiffness mapping image of tri-dome shown in **c**. The layer-dependent stiffness can be clearly distinguished.

In a bi-dome system, three joint angles between adjoining edges were formed, termed as *α*_1_, *α*_2_ and *α*_3_ (inset in Fig. 5a). The values of *α*_1_, *α*_2_ and *α*_3_ for WS_2_ bi-domes were measured to be 153.1° ± 8.8°, 75.6° ± 9.8° and 131.2° ± 6.1°, respectively (Fig. 5a), differing with each other, which is in great contrast to Plateau’s law of equal joint angles of 120° as observed in soap bubbles^1^. The values of *α*_1_, *α*_2_ and *α*_3_ for WS_2_ bi-domes were calculated (Supplementary Note 8 and Supplementary Fig. 10) to be 152.6°, 72.3° and 135.1°, respectively, agreeing well with the experimental values (Fig. 5a). A large difference of ~77.5° between *α*_1_ and *α*_2_ was observed in WS_2_ bi-dome systems, such large deviation has not been previously reported, demonstrating a variant Plateau’s law. The governing reason for this behaviour is due to the large unequal effective surface tension (*σ*) differences in the different dome layers, where 1 L, 2 L and 3 L domes have experimental *σ* values of 2.4 ± 0.3, 3.5 ± 0.3, 3.6 ± 0.3 N/m, respectively, which is significantly higher than other systems (Supplementary Note 8). The large difference among the *σ* values of 1 L, 2 L and 3 L domes is a direct result of the discrete nature of *γ* in 1–3 L TMDs as shown in Fig. 3b. Additionally, some domes in this work are partially sitting on top of others; the dome part sitting on top of another dome has different *γ* compared to the part being exfoliated from the bulk flake. For example, it requires less energy to detach a 1 L sheet from a 2 L sheet, rather than from the bulk (Supplementary Fig. 10b). Domes merging in this manner demonstrate the discrete nature of *γ*, leading to significant variations in the effective *σ* of each dome. Similarly, large variations of the joint angles were also observed and analytically calculated for MoS_2_, demonstrating excellent agreement (Supplementary Fig. 11a).

To exacerbate the large deviations from Plateau’s law in TMD domes, the joint angles *β*_1,_ *β*_2_ and *β*_3_ of WS_2_ tri-dome systems were also experimentally determined as shown in Fig. 5b–d. The values of *β*_1,_ *β*_2_ and *β*_3_ for WS_2_ tri-domes were measured to be 135.5° ± 6.4°, 104.6° ± 7.1° and 119.9° ± 3.7°, respectively, also clearly demonstrating variant Plateau’s law, whereby joint angles no longer merge at 120°. Similar large angle deviations were also observed in MoS_2_ tri-domes (Supplementary Fig. 11b). The largest angle was found in the 1 L region in a tri-dome system, similar to that observed in a bi-dome system; the angle difference (*β*_1_ - *β*_2_) in a tri-dome system is smaller than that in a bi-dome system (*α*_1_ - *α*_2_). A statistical analysis of angle variation in WS_2_ and MoS_2_ bi-dome and tri-dome networks (Fig. 5a, b, and Supplementary Fig. 11) concluded that *α*_1_ and *β*_1_ for both material systems (WS_2_ and MoS_2_) are the largest among all three angles. Moreover, bi-dome systems have larger angle deviation compared to tri-dome systems. The *α*_1_, *α*_2_ and *α*_3_ of a WS_2_ bi-dome system deviate by 8.8°, 9.8° and 6.1°, respectively, whilst *β*_1_, *β*_2_ and *β*_3_ exhibit lower deviations of 6.4°, 7.1° and 3.7°, respectively. This could be attributed to the fact that bi-domes are more likely to be influenced by the surrounding conditions on the bulk flake, including side domes, surface contaminants and surface topology, leading to larger deviation in joint angles *α*. Owing to a trigonal structure in tri-dome systems, a better structural stability generates smaller deviation in joint angles compared to bi-domes, and WS_2_ and MoS_2_ tri-dome systems have comparable angle values for *β*_1_, *β*_2_ and *β*_3_.

In addition, it is worth to note that the validity of the variant of Plateau’s law in complex dome network systems. To prove this, we characterised a one-dimensional (1D) dome chain and 2D superlattice structure with bi-dome and tri-domes as the repeating unit (Supplementary Fig. 12). In the 1D dome chain, it also proves that the largest and smallest angles are *α*_1_ in 1 L regions and *α*_2_ in 2 L regions, respectively, which is confirmed by stiffness mapping (Supplementary Fig. 12b). This reaffirms that the variations in joint angles, regardless of the complexity of the network structure, are induced by layer-dependent mechanical properties including *E*_2D_, *γ* and *σ*. Furthermore, due to the robustness of TMDs, *E*_2D_ and *γ* remain constant over time, allowing the morphological structure and joint angles to endure for a long period without variations (Supplementary Fig. 13), even in the presence of external stimuli (nano-indentation and optical tests). This means determination of joint angles of the network is a reliable tool to quickly identify layer number of domes in a network, exemplified by the 2D dome network shown in Supplementary Fig. 12c.

We successfully fabricated 1 L, 2 L and 3 L pressurised hydrogen nano-domes, and their networks, using low-energy proton irradiation on TMD flakes. Dome layer number was identified and confirmed by optical contrast, SHG imaging and high-resolution stiffness mapping. The layer-dependent mechanical properties of domes revealed by AFM nano-indentation match well with FEA simulations. 1–3 L domes provide a fascinating platform for resolving the discrete layer-dependent adhesion energies in TMDs, which matched with DFT calculations. Joint bi-dome and tri-dome systems were formed when the domes sitting in different basal planes grew and interacted with each other. The layer-structure and configurations of joint domes were successfully identified and confirmed by stiffness mapping, FEA calculation and bursting test. Variant Plateau’s law was observed in bi-dome and tri-dome systems, which is due to the large variations of effective surface tension in different layer number domes, arising from the discrete vdWs adhesion energy in TMDs. For example, our bi-dome system has a large joint angle difference of more than 77°, in great contrast to conventional liquid bubble systems that have equal joint angles. Our solid domes equivalent surface tension values range from 2.4 to 3.6 N/m, which is two orders of magnitude larger than their liquid or foam counterparts enabling high pressure encapsulation. The variant Plateau’s law observed in experiments agrees with our analytical calculation. Furthermore, stable and long-lasting 1D and 2D dome networks were realised in experiments with multiple domes sitting in different basal planes of the TMD. Dome networks can become a promising topic for generating 1D or 2D nanostructures with large variation in properties enabling new device applications in the fields of nano-photonics, nano-opto-mechanics and quantum science.

## Methods

### Fabrication of the domes

Thick TMD flakes are mechanically exfoliated onto various substrates including SiO_2_/Si (275 nm SiO_2_), Au deposited and doped silicon. The samples are subsequently placed in the chamber for proton irradiation treatment in a high vacuum condition. During the treatment, the pressure inside the chamber is *P* = 1.4 × 10^−4^ mbar, with hydrogen gas delivered at a flow rate of 30 sccm. Meanwhile, the H^+^ protons produced in an ionisation chamber will be accelerated and guided onto the sample surface, as a form of proton beam with energy at least 25 eV. The relative larger energy enables protons to deliberately penetrate deeper basal planes of the TMD, creating different layered domes and dome networks. The entire treatment process normally takes a few hours to complete.

### Optical characterisation

Optical microscope images were taken by a Zeiss 780 confocal microscope equipped with a 633 nm single photon laser. SHG measurement were also performed on a Zeiss 780 confocal microscope using a Ti:Sapphire laser (150 fs, 80 MHz). The sample is excited and measured under a 50× confocal objective lens (NA = 0.85), and results are collected in the reflection mode at a fundamental laser wavelength of 900 nm. All data of optical characterisation measurements were processed and analysed by image processing software, Zen 3.2 (blue edition).

### AFM Nano-indentation

The topographic images were captured using a Bruker Dimension Icon AFM. The aspect ratio of the domes was measured in Scanasyst mode with soft cantilevers, Scanasyst-Air, whose nominal spring constant *k* and nominal tip radius are 0.4 N/m and 2 nm, respectively. Using soft tips to obtain the profile and topographic information is designed to minimise tip–sample interaction and avoid modifying the shape of the domes or destroy domes. Indentation experiments on TMD domes were performed in Quantum Nano Mechanical (QNM) mode. The tips used for the measurement is RTESPA-300 with *k* = 40 N/m and a nominal *R*_*tip*_ = 8 nm. Before the measurement, the tips required calibration in terms of resonance frequency, deflection sensitivity, contact area, and spring constant to ensure results are repeatable. The indentation force was adjusted and set between 50 and 100 nN to avoid the fact that large forces might destroy domes. For the force curves at different locations, they were extracted using ramp mode and this mode could record extending and extracting cycles at different locations on the dome samples. Stiffness values could be processed by calculating the slope of the loading part of extending cycles. For the adhesion energy measurement, the 2D modulus (*E*_2D_) was extracted and calculated based on the force curves at the centre of the dome samples.

## Supplementary information

Supplementary Information

## Data Availability

The authors declare that all data supporting the finding could be found in the manuscript and supporting information of this work. The datasets generated during the current study are available upon request from the corresponding author.

## References

[1] Plateau, J. A. F. *Statique expérimentale et théorique des liquides soumis aux seules forces moléculaires*. Vol. 2 (Gauthier-Villars, 1873).

[2] Kraynik AM, Reinelt DA, van Swol F (2004). Structure of Random Foam. Phys. Rev. Lett..

[3] Taylor JE (1976). The Structure of singularities in soap-bubble-like and soap-film-like minimal surfaces. Ann. Math..

[4] Weaire D, Phelan R (1994). A counter-example to Kelvin’s conjecture on minimal surfaces. Philos. Mag. Lett..

[5] Almgren, F. J. & Taylor, J. E. The Geometry of Soap Films and Soap Bubbles. *Scientific American* **235**, 82–93 (1976).

[6] Hutchings M, Morgan F, Ritoré M, Ros A (2002). Proof of the double bubble conjecture. Ann. Math..

[7] Araújo H (2018). The effect of geometry on the flexural properties of cellular core structures. Proc. Inst. Mech. Eng., Part L: J. Mater.: Des. Appl..

[8] Chuang C-H, Huang J-S (2002). Elastic moduli and plastic collapse strength of hexagonal honeycombs with plateau borders. Int. J. Mech. Sci..

[9] Simone AE, Gibson LJ (1998). Effects of solid distribution on the stiffness and strength of metallic foams. Acta Materialia.

[10] Aquino J, Duarte I, Dias-de-Oliveira J (2018). Modelling and effective properties prediction of metal foams. Sci. Technol. Mater..

[11] Novoselov KS (2004). Electric field effect in atomically thin carbon films. science.

[12] Stavans J, Glazier JA (1989). Soap froth revisited: dynamic scaling in the two-dimensional froth. Phys. Rev. Lett..

[13] Weaire D (1990). Comment on “Soap froth revisited: Dynamical scaling in the two-dimensional froth”. Phys. Rev. Lett..

[14] Glazier JA, Gross SP, Stavans J (1987). Dynamics of two-dimensional soap froths. Phys. Rev. A.

[15] Stine KJ, Rauseo SA, Moore BG, Wise JA, Knobler CM (1990). Evolution of foam structures in Langmuir monolayers of pentadecanoic acid. Phys. Rev. A.

[16] Neimark AV, Vignes-Adler M (1995). Variations from the Plateau law in foams. Phys. Rev. E.

[17] Hauner IM, Deblais A, Beattie JK, Kellay H, Bonn D (2017). The Dynamic Surface Tension of Water. J. Phys. Chem. Lett..

[18] Román FL, Faro J, Velasco S (2001). A simple experiment for measuring the surface tension of soap solutions. Am. J. Phys..

[19] Radisavljevic B, Radenovic A, Brivio J, Giacometti V, Kis A (2011). Single-layer MoS_2_ transistors. Nat. Nanotechnol..

[20] Chen P (2021). Approaching the intrinsic exciton physics limit in two-dimensional semiconductor diodes. Nature.

[21] Khestanova E, Guinea F, Fumagalli L, Geim A, Grigorieva I (2016). Universal shape and pressure inside bubbles appearing in van der Waals heterostructures. Nat. Commun..

[22] Jia P (2019). Programmable graphene nanobubbles with three-fold symmetric pseudo-magnetic fields. Nat. Commun..

[23] He L (2019). Isolating hydrogen in hexagonal boron nitride bubbles by a plasma treatment. Nat. Commun..

[24] Lloyd D (2017). Adhesion, stiffness, and instability in atomically thin MoS_2_ bubbles. Nano Lett..

[25] Tedeschi D (2019). Controlled Micro/Nanodome Formation in Proton‐Irradiated Bulk Transition‐Metal Dichalcogenides. Adv. Mater..

[26] Koenig SP, Boddeti NG, Dunn ML, Bunch JS (2011). Ultrastrong adhesion of graphene membranes. Nat. Nanotechnol..

[27] Mak KF, Lee C, Hone J, Shan J, Heinz TF (2010). Atomically Thin MoS_2_: A new direct-gap semiconductor. Phys. Rev. Lett..

[28] Novoselov KS (2005). Two-dimensional atomic crystals. P Natl Acad. Sci. USA.

[29] Afanasyev YD, Andrews GT, Deacon CG (2011). Measuring soap bubble thickness with color matching. Am. J. Phys..

[30] Falin A (2021). Mechanical properties of atomically thin Tungsten Dichalcogenides: WS_2_, WSe2, and WTe2. ACS Nano..

[31] Bertolazzi S, Brivio J, Kis A (2011). Stretching and Breaking of Ultrathin MoS_2_. ACS Nano..

[32] Chen Z (2011). Core–shell MoO_3_–MoS_2_ Nanowires for Hydrogen Evolution: A functional design for electrocatalytic materials. Nano Lett..

[33] Miyake K, Shigekawa H (1995). Surface structures of layered compounds treated with alkali-metal hydroxide solutions studied by scanning tunneling microscopy. Synth. Met..

[34] Li X, Zhu H (2015). Two-dimensional MoS_2_: Properties, preparation, and applications. J. Materiomics.

[35] Li H (2012). Optical Identification of Single- and Few-Layer MoS_2_ Sheets. Small.

[36] Wu W (2014). Piezoelectricity of single-atomic-layer MoS_2_ for energy conversion and piezotronics. Nature.

[37] Zhu H (2015). Observation of piezoelectricity in free-standing monolayer MoS_2_. Nat. Nanotechnol..

[38] Vella D, Davidovitch B (2017). Indentation metrology of clamped, ultra-thin elastic sheets. Soft Matter.

[39] Di Giorgio C (2020). Nanoscale measurements of elastic properties and hydrostatic pressure in H2-Bulged MoS_2_ Membranes. Adv. Mater. Interfaces.

[40] Rokni H, Lu W (2020). Direct measurements of interfacial adhesion in 2D materials and van der Waals heterostructures in ambient air. Nat. Commun..

[41] Wang W (2015). Measurement of the cleavage energy of graphite. Nat. Commun..

[42] Koren E, Lörtscher E, Rawlings C, Knoll AW, Duerig U (2015). Adhesion and friction in mesoscopic graphite contacts. Science.

[43] Li B (2019). Probing van der Waals interactions at two-dimensional heterointerfaces. Nat. Nanotechnol..

[44] Blundo E, Yildirim T, Pettinari G, Polimeni A (2021). Experimental adhesion energy in van der waals crystals and heterostructures from atomically thin bubbles. Phys. Rev. Lett..

[45] Aitken ZH, Huang R (2010). Effects of mismatch strain and substrate surface corrugation on morphology of supported monolayer graphene. J. Appl. Phys..

[46] Zhao Z-Y, Liu Q-L (2018). Study of the layer-dependent properties of MoS_2_ nanosheets with different crystal structures by DFT calculations. Catal. Sci. Technol..

[47] Medhekar NV, Ramasubramaniam A, Ruoff RS, Shenoy VB (2010). Hydrogen bond networks in graphene oxide composite paper: Structure and mechanical properties. ACS Nano..

