# Supplementary material for: Variant Plateau’s law in atomically thin transition metal dichalcogenide dome networks
Source: Nat Commun. 2023 Feb 24;14:1050. doi: 10.1038/s41467-023-36565-2 (PMC9958105; doi:10.1038/s41467-023-36565-2)
Supplement: Supplementary file 1 — Supplementary Information [file 41467_2023_36565_MOESM1_ESM.pdf]

**Supplementary Information for**

# Variant Plateau's Law in Atomically Thin Transition Metal Dichalcogenide Dome Networks

Boqing Liu[1†], Tanju Yildirim[2†], Tieyu Lü[3], Elena Blundo[4], Li Wang[5], Lixue Jiang[6], Hongshuai Zou[7], Lijun Zhang[7], Huijun Zhao[6], Zongyou Yin[8], Fangbao Tian[5], Antonio Polimeni[4], Yuerui Lu[1,9*]

[1]School of Engineering, College of Engineering and Computer Science, The Australian National University, Canberra, ACT 2601, Australia

[2]Center for Functional Sensor & Actuator (CFSN), Research Center for Functional Materials, National Institute for Materials Science (NIMS), 1-1 Namiki, Tsukuba, Ibaraki 305-0044, Japan

[3]Department of Physics and Institute of Theoretical Physics and Astrophysics, Xiamen University, Xiamen, 361005, China

[4]Dipartimento di Fisica Sapienza Università di Roma, 00185 Roma, Italy

[5]School of Engineering and Information Technology, University of New South Wales, Canberra, ACT 2600, Australia

[6]Centre for Catalysis and Clean Energy, Gold Coast Campus, Griffith University, Queensland 4222, Australia

[7]State Key Laboratory of Integrated Optoelectronics, School of Materials Science and Engineering, and Jilin Provincial International Cooperation Key Laboratory of High-Efficiency Clean Energy Materials, Jilin University, Changchun 130012, China

[8]Research School of Chemistry, College of Science, The Australian National University, Canberra, ACT 2601, Australia

[9]ARC Centre of Excellence in Quantum Computation and Communication Technology ANU node, Canberra, ACT 2601, Australia

[†]These authors equally contributed to this article

*****To whom correspondence should be addressed: Yuerui Lu (yuerui.lu@anu.edu.au)

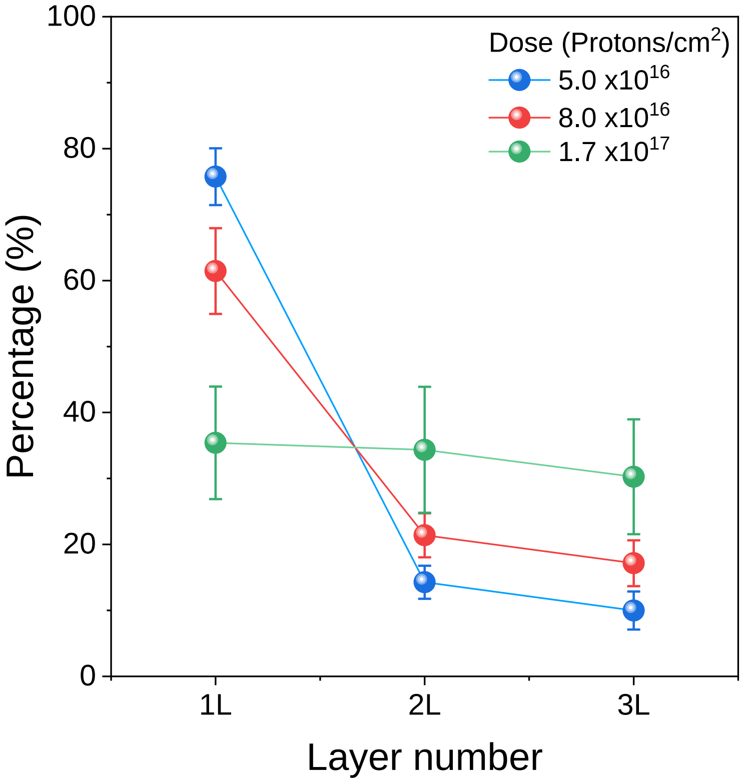


**Supplementary Figure 1 |** Formation yield percentage of 1-3L $WS_2$ bubbles as a function of proton irradiation dosage. The data was collected from $WS_2$ flakes on $SiO_2$ substrates. For each dosage level, more than 150 domes were counted.

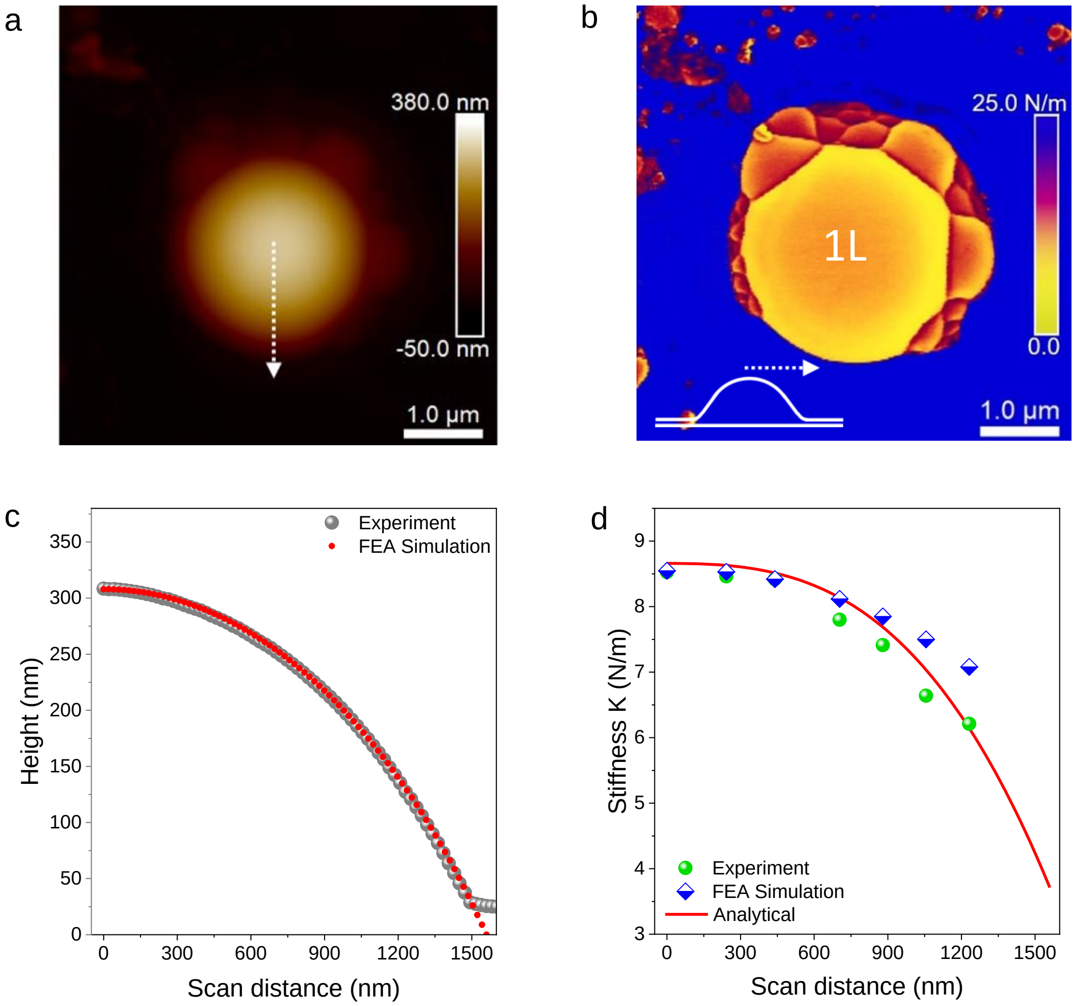


**Supplementary Figure 2 | Mechanical stiffness of a single layered $WS_2$ dome. a**, AFM image of a large $WS_2$ monolayer dome. **b**, Stiffness mapping of the 1L $WS_2$ dome shown in (a). The inset indicates the schematic of cross section of the dome. **c**, Measured height profile (grey) of the 1L $WS_2$ dome along the white dashed line shown in **a**. The simulated profile (red) generated by FEA well matches with the measured one. **d**, Measured stiffness (solid green dots) as a function of scan distance along the white dashed line shown in **a.** As shown in the figure, there is large stiffness variation along the dome with the largest stiffness observed at the centre and reduces towards the edge. Simulated stiffness values by FEA calculation (blue-white diamond) and analytical method (red solid line) (see Supplementary Note 7 and Eq. (S25) for the analytical solution), match reasonably well with experimental values.

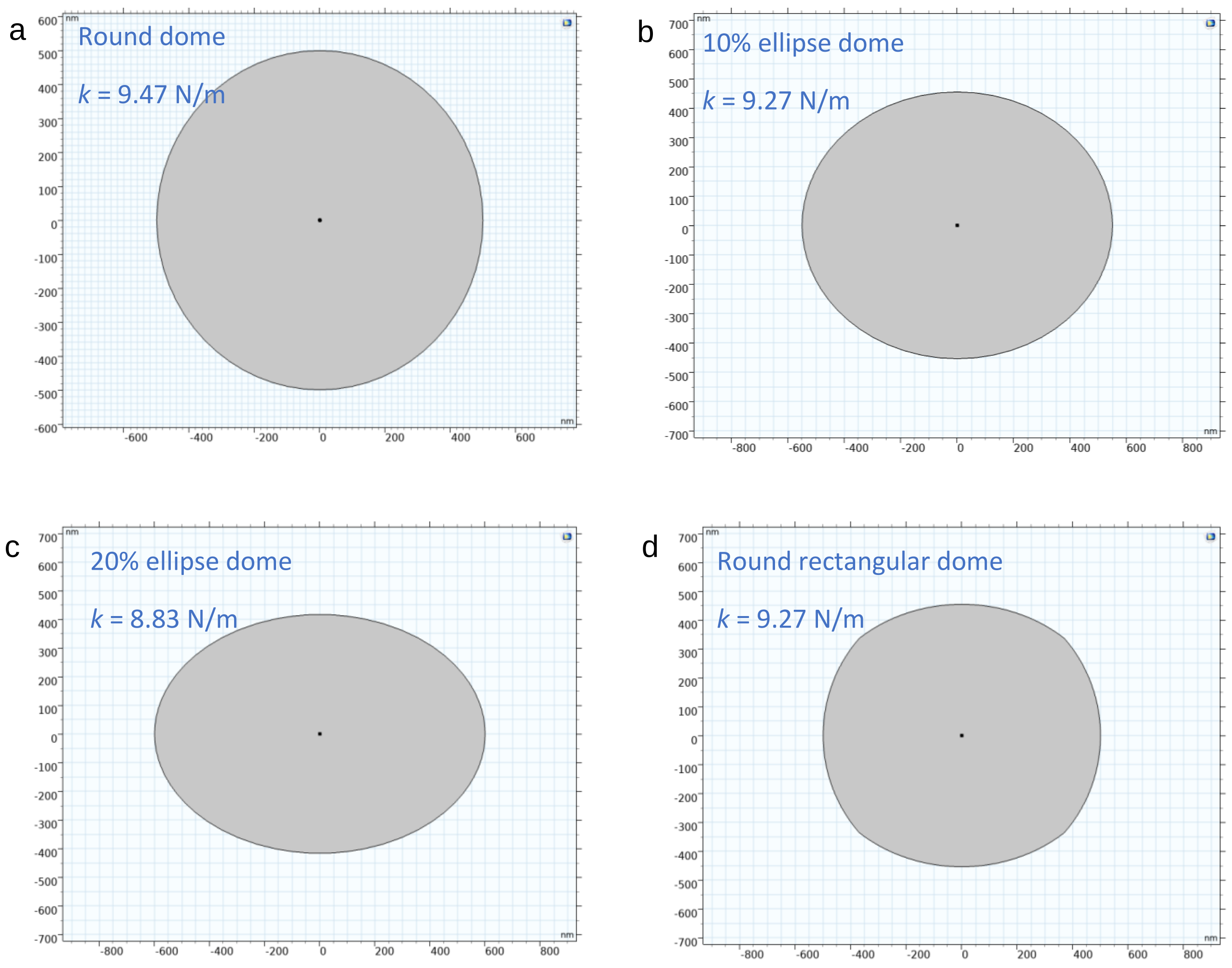


**Supplementary Figure 3 | Simulated stiffness values of domes with different footprint shapes and an internal pressure of 80 atm. a**, Perfectly round circular dome with a footprint radius = 500 nm results in a stiffness value ($k$) of 9.47 N/m **b**, An ellipse shape dome with 10% increment for the length in x-axis and 10% decrement for length in y-axis gives $k$ = 9.27 N/m. **c**, An ellipse shape dome with 20% elongation of the length in x-axis and 20% decrement for length in y-axis gives $k$ = 8.83 N/m. **d**, Dome with round rectangular edges, whose length in x axis is 1000 nm and length in y axis is 900nm gives $k$ = 9.27 N/m.

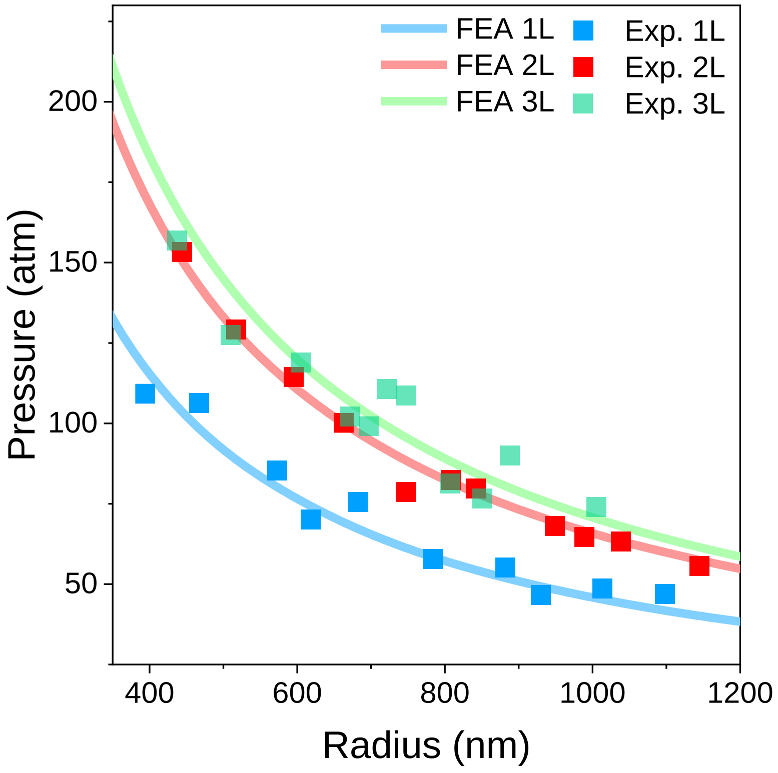


**Supplementary Figure 4 |** Dome pressure as a function of dome radius for 1-3L domes, from both experiments and FEA calculations. Solid boxes are extracted values from height profiles and stiffness values measured from AFM nano-indentation, while dashed lines are calculation results from FEA. $h_m/R$ values of 1-3L domes were measured to be 0.18, 0.17 and 0.16, respectively, which were also used in FEA calculations.

a

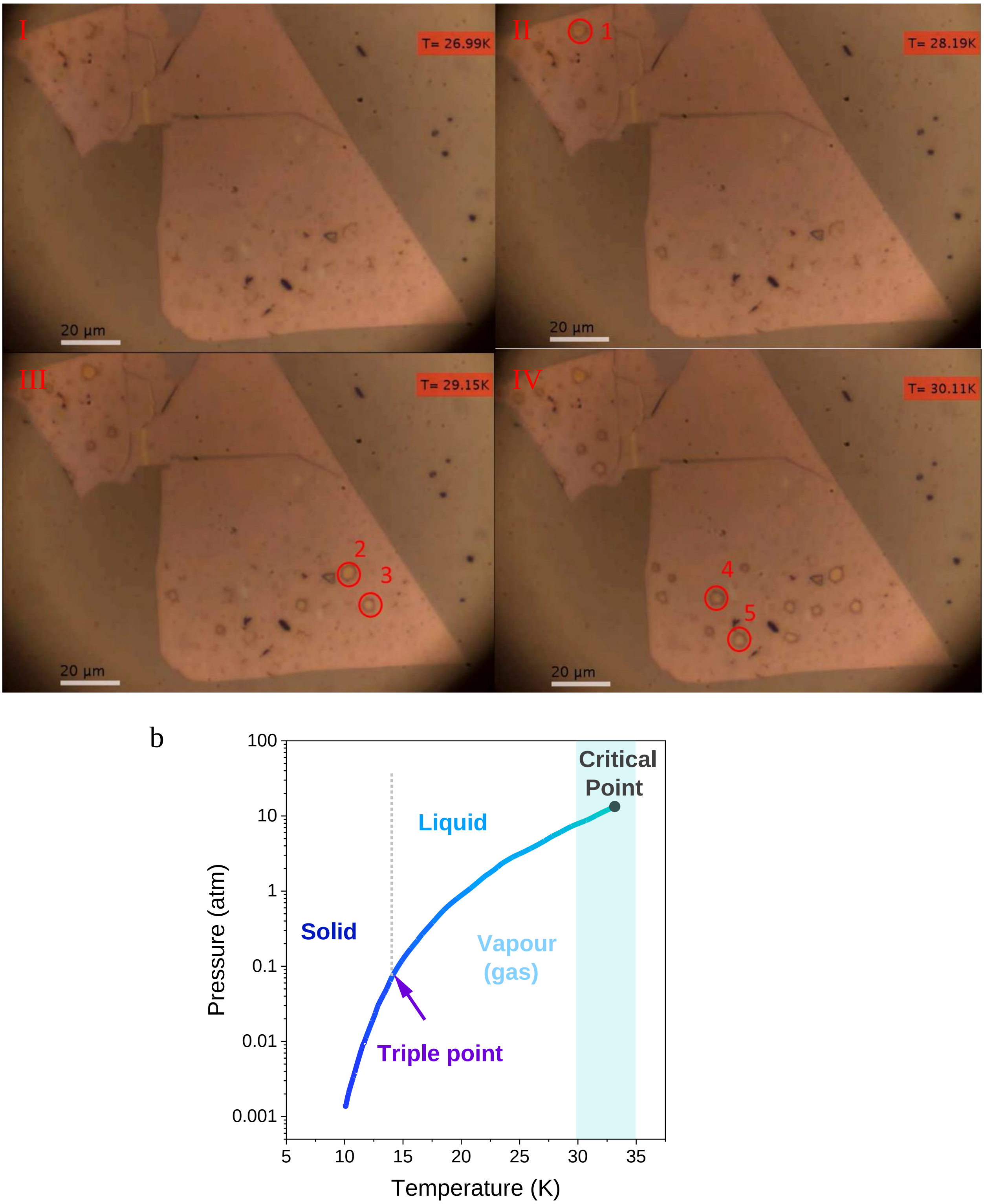


**Supplementary Figure 5 | Characterisation of gas pressure in domes *via* low temperature method. a,** Optical microscopic images of 1L $WS_2$ dome evolution with temperature. All domes deflated at 27 K (I), and then the first dome inflated at 28 K (II). Dome 2 and 3 formed at 29 K (III), and smaller domes 4 and 5 inflated at 30 K (IV), Panel **a** adapted with permission from ref.[9], Copyright 2019, Wiley-VCH. **b,** Pressure-temperature (p -T) phase diagram of hydrogen. Blue curve marks off the solid and liquid (left) and vapor (right) phases. The vertical-coloured bar represents the range of phase transition temperature $T^{H}{}_{LV}$ values for $WS_2$ domes.

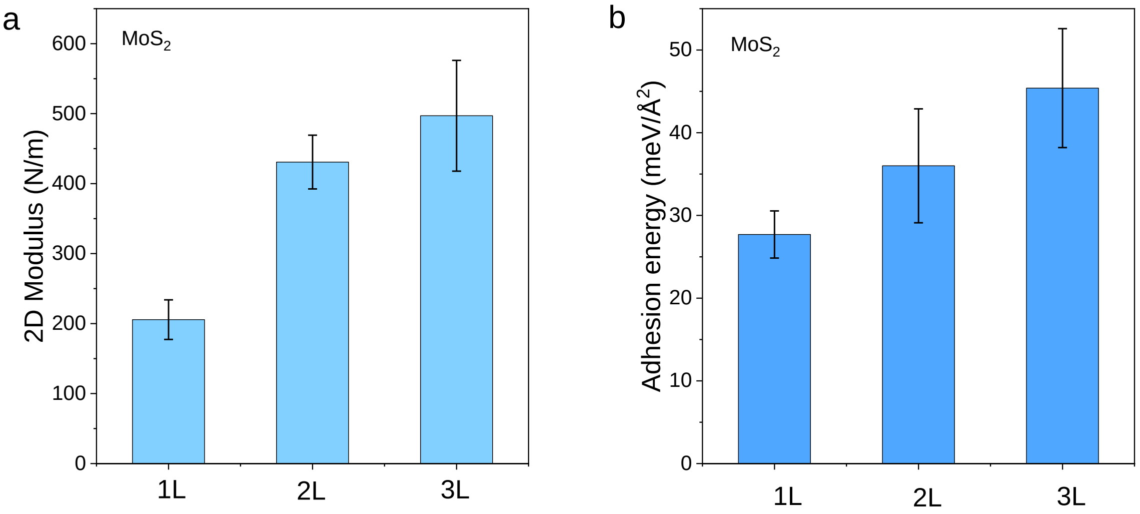


**Supplementary Figure 6 | Characterisation of 1-3L $MoS_2$ domes.** **a**, Extracted two-dimensional modulus ($E_{2D}$) of $MoS_2$ as a function of layer number. The extracted $E_{2D}$ values are 205.6±28.3, 430.8±38.2 and 496.9±79.2 N/m for 1-3L $MoS_2$ domes, respectively. **b**, Measured layer-dependent adhesion energies of $MoS_2$, which are the energies required to exfoliate top layers (1-3L) from a $MoS_2$ bulk flake, using nano-domes. The adhesion energy values obtained from 1-3L $MoS_2$ are 27.7±2.9, 36.16.8 and 45.5 ± 7.1 meV/Å$^2$, respectively. The error bars in **a** and **b** represent statistical variation from at least 15 domes for each group with different layer number.

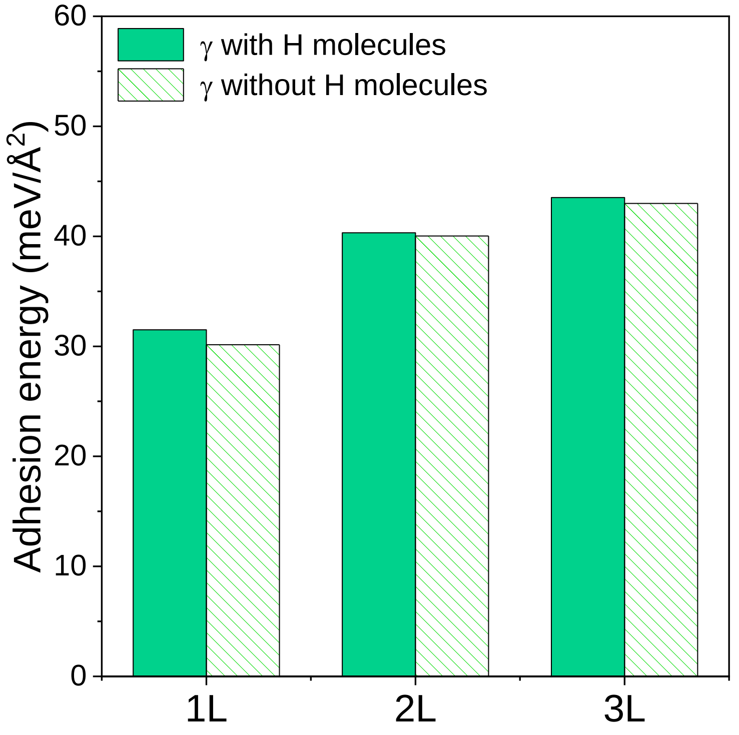


**Supplementary Figure 7 | Effect of hydrogen molecule adsorption on TMD properties.** DFT calculation of $WS_2$ layer dependent adhesion energy (dash) compared with the enhanced values induced by hydrogen molecule adsorption (solid).

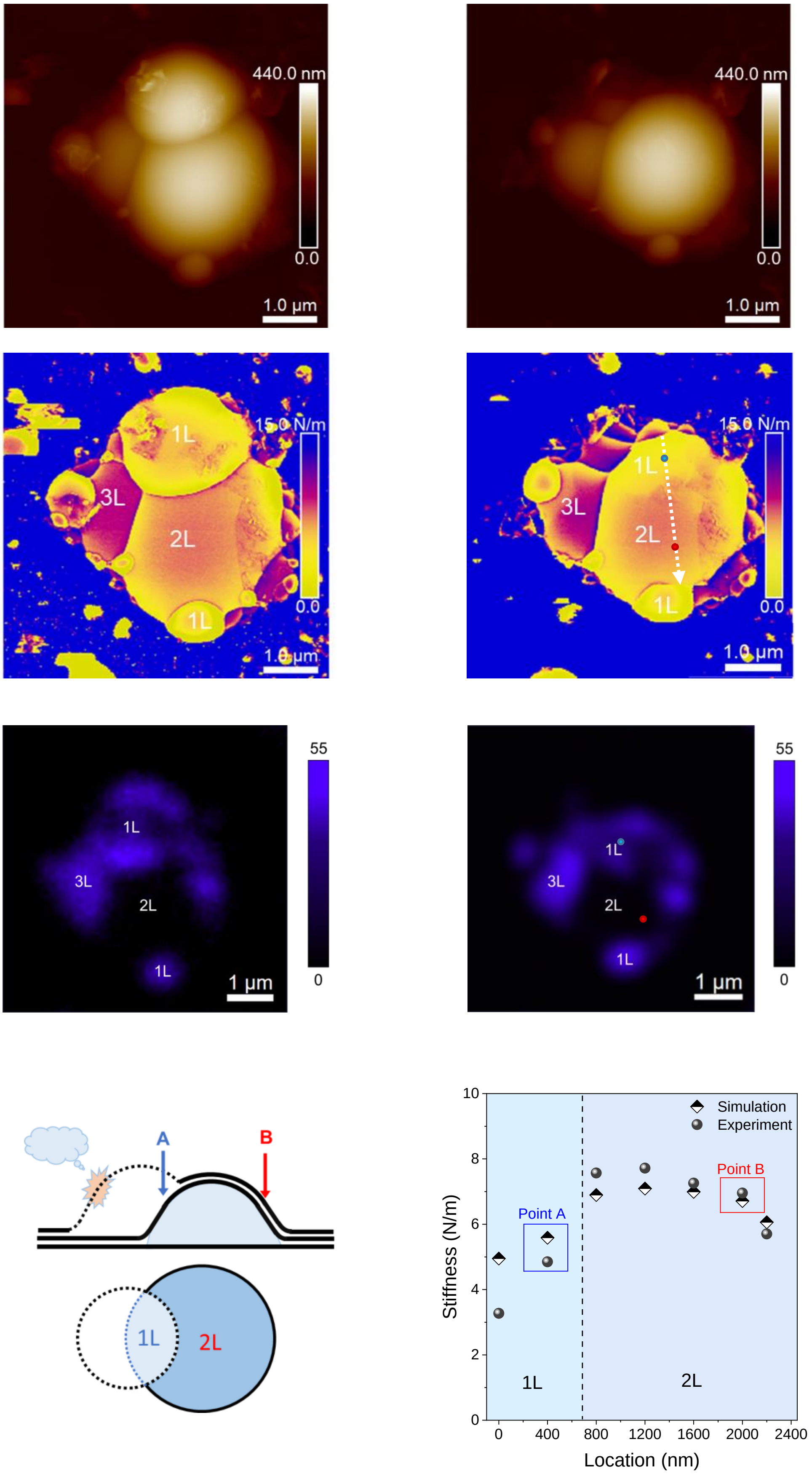
440.0 nm
0.0
1.0 µm
440.0 nm
0.0
1.0 µm
1L
3L
2L
1L
15.0 N/m
0.0
1.0 µm
1L
3L
2L
1L
15.0 N/m
0.0
1.0 µm
55
0
1L
3L
2L
1L
1 µm
55
0
1L
3L
2L
1L
1 µm
A
B
1L
2L
Simulation
Experiment
Point A
Point B
Stiffness (N/m)
Location (nm)
1L
2L
0
2
4
6
8
10
0
400
800
1200
1600
2000
2400

**Supplementary Figure 8 | Bursting test to confirm the configuration of a joint bi-dome.** **a-c**, AFM (**a**), stiffness mapping (**b**) and SHG mapping (**c**) images of a joint tri-dome. The 1L, 2L and 3L domes can be clearly assigned based on the stiffness and SHG images. **d-f**, AFM (**d**), stiffness mapping (**e**) and SHG mapping (**f**) images of the same area shown in **a-c**, when the 1L dome was deliberately burst by using AFM indentation, while 2L and 3L domes survived. After the bursting of the 1L dome, a 2L region (B region in **e**) and a 1L region (A region in **e**) coexist over the 2L dome's surface simultaneously. **g**, Schematics showing the configuration of the joint bi-dome, after the 1L dome was burst; the top plot is the side view, and the bottom is the top view; the solid line and dash line represent the remaining dome and bursting 1L dome; points A and B are the locations shown in **e**. **h**, Measured stiffness (solid black dots) as a function of scan distance along the white dashed line shown in **e**. Simulated stiffness values by FEA calculation (diamond) reasonably match with the experimental values. The vertical dashed line represents the boundary between A and B regions in **e**.

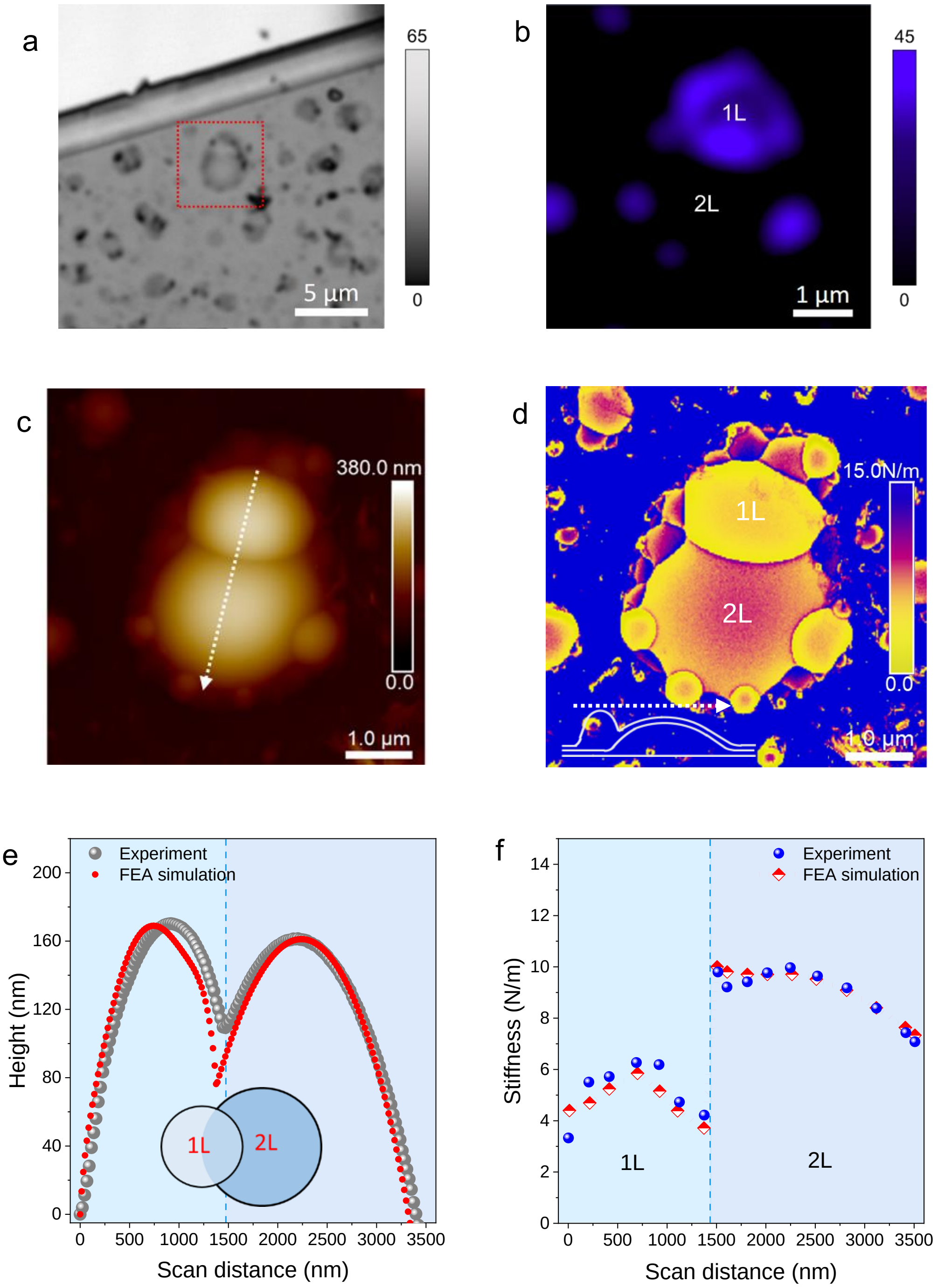


**Supplementary Figure 9 │ Identification of structure configuration of a joint bi-dome with larger 2L dome *via* nano-indention. a**, Optical microscope image of $WS_2$ domes formed on top of a TMD flake. The red dashed box highlights a joint bi-dome that is composed of a large 2L dome and a smaller 1L dome. The colour of the 1L dome is light grey, but the 2L dome's colour is darker with increasing layer number due to optical contrasts in membrane thickness. **b**, SHG mapping of the joint bi-dome, as shown by the red dashed box area in **a**; therein, the 1L dome showed strong SH emission while the 2L dome was not detectable under the same excitation and collection conditions due to the inversion symmetry in 2L $WS_2$. **c**, AFM image of the joint $WS_2$ bi-dome shown in the red dashed box in **a**. **d**, Stiffness mapping

of the joint bi-dome shown in **c**. The inset indicates the structure of the domes along the white dashed line in **c**. **e**, Measured height profile (grey) of the joint bi-dome along the white dashed line shown in **e**. The simulated profile (red) generated by FEA reasonably matches with the measured one. **d**, Measured stiffness (solid blue dots) as a function of scan distance along the white dashed line shown in **c**. **e**, Simulated stiffness values by FEA calculation (red diamond) well match with the experimental values. The sharp drop of stiffness value at 1500 nm, which corresponds to the joint boundary between 2L and 1L domes.

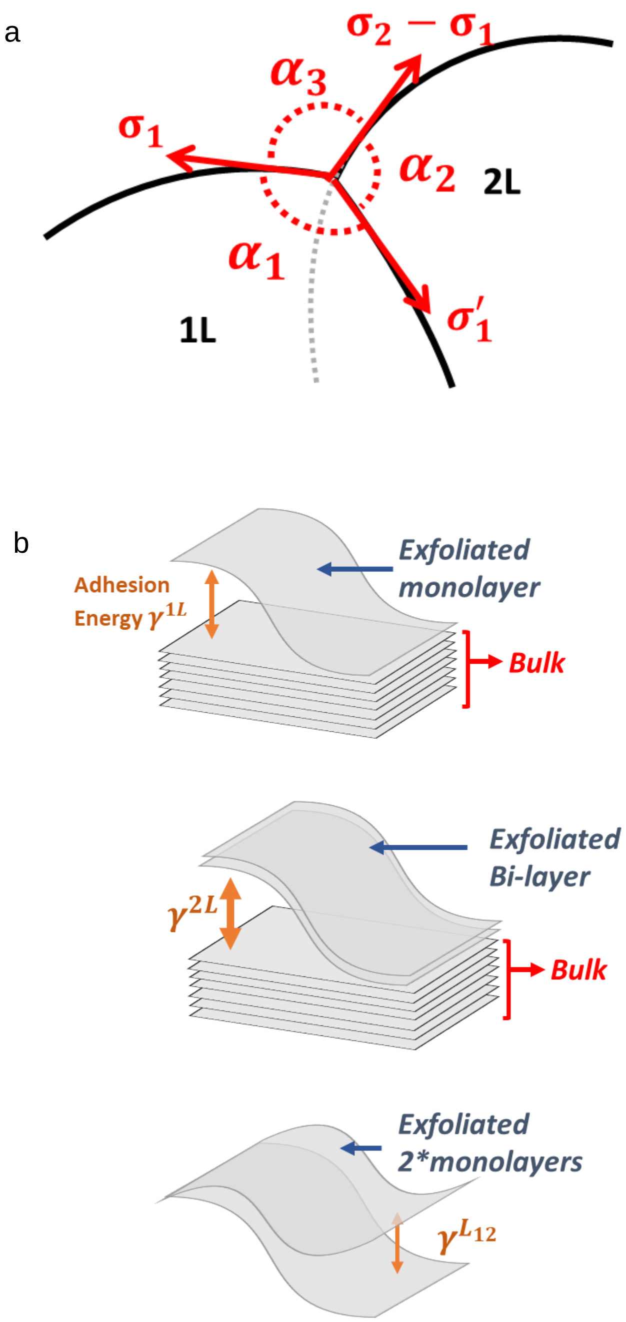


**Supplementary Figure 10 | Deriving the angles for variant Plateau's law. a**, Force equilibrium for calculating joint angles. $\sigma$ represent surface tension. $\sigma$ values and their meanings are given in Supplementary Note 6. **b**, Schematics showing discrete energies $\gamma$ required for exfoliating layers in different scenarios, which are given in Supplementary Note 6. $\gamma^{1L}$ and $\gamma^{2L}$ are the adhesion energies of 1L and 2L TMD, respectively. $\gamma^{L_{12}}$ is the energy required for the exfoliation of a top single layer from a bilayer TMD.

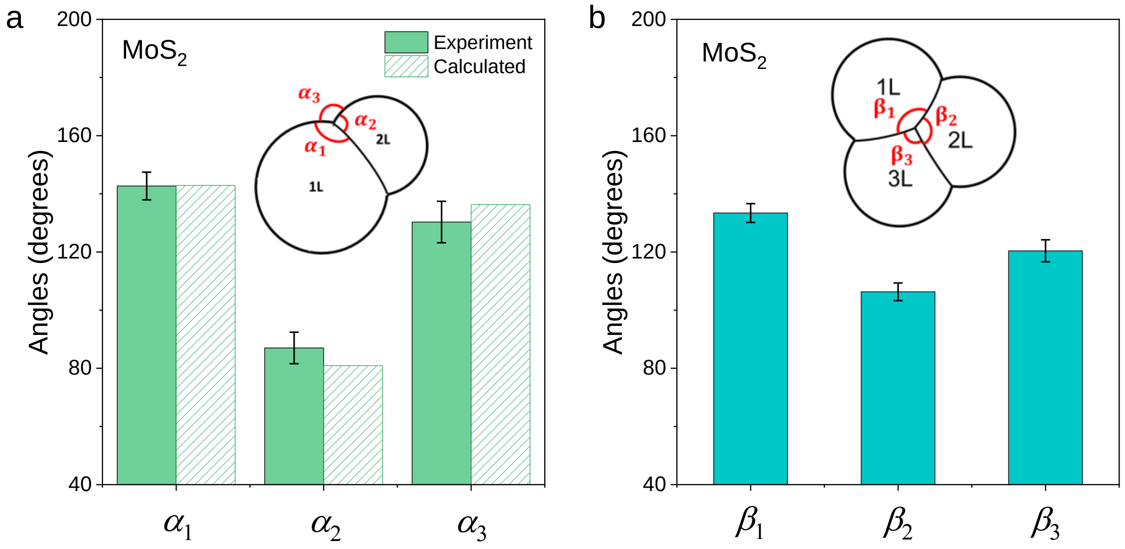


**Supplementary Figure 11 | Variant Plateau's law in bi- and tri-dome $MoS_2$ systems. a**, Histogram of the joint angles ($\alpha_i$, $i$ = 1, 2, 3) in $MoS_2$ bi-dome systems, extracted experimentally (solid bars) and analytically (patterned bars). The inset shows the angle notations in a standard bi-dome configuration. The experimental values for joint angle are 142.7º±4.6º, 87.0º±5.4º and 130.3º±7.2º for $\alpha_1$, $\alpha_2$ and $\alpha_3$, respectively. Statistical data was collected from 20 joint bi-domes. The calculated values for $\alpha_1$, $\alpha_2$ and $\alpha_3$, are 142.9º, 80.9º and 136.3º, respectively, all of which are in a good agreement with experimental values. **b**, Histogram of the joint angles ($\beta_i$, $i$ =1, 2, 3) in $MoS_2$ tri-dome systems, extracted experimentally. The inset shows the angle notations in a standard tri-dome configuration. The experimental values for joint angle are 133.4º±3.3º, 106.3º±3.0º and 120.3º±4.0º for $\beta_1$, $\beta_2$ and $\beta_3$, respectively. Statistical data was collected from 15 joint tri-domes.

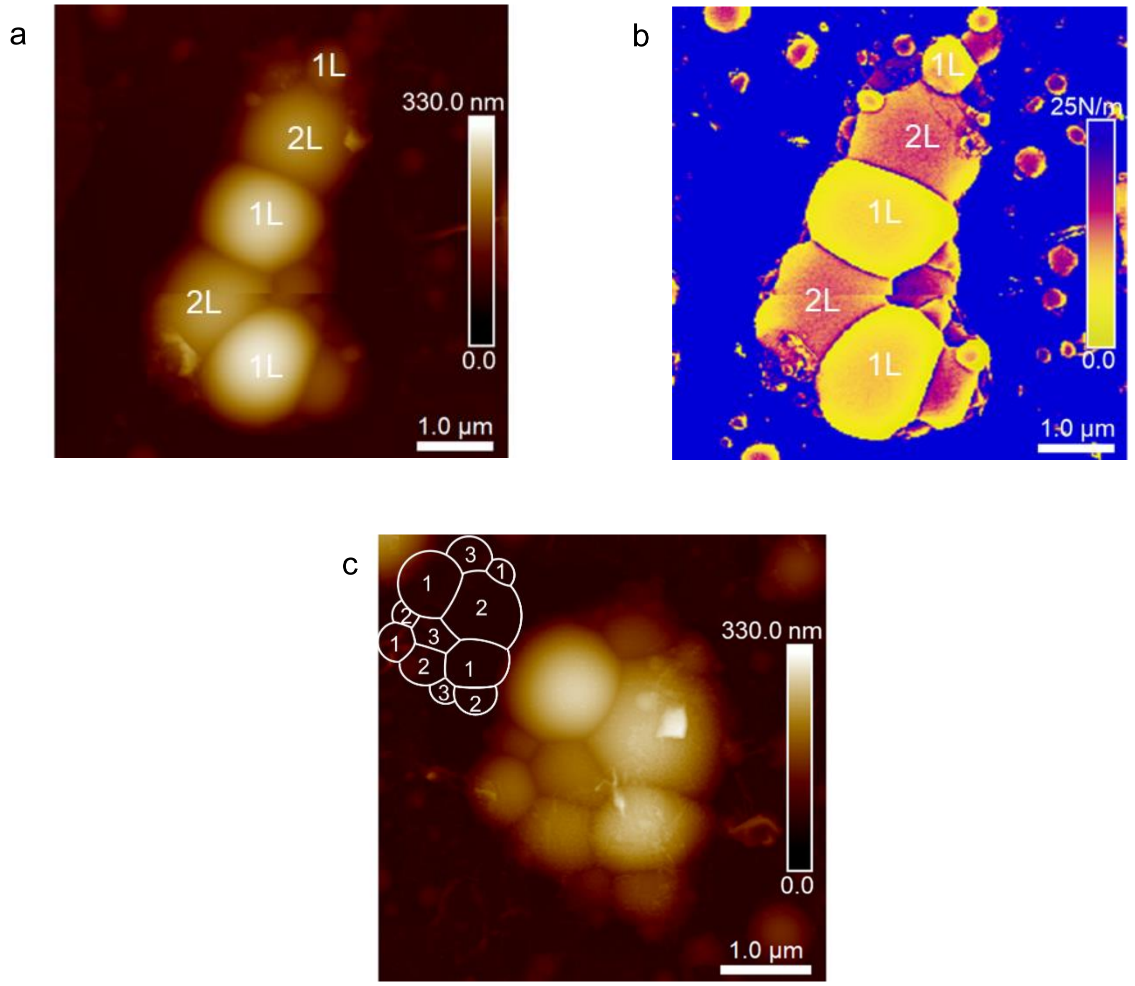


**Supplementary Figure 12 | 1D and 2D nano-dome networks. a**, AFM image of a 1D nano-dome chain with repeated 1L and 2L bi-dome units. **b**, Stiffness mapping image of the 1D nano-dome chain shown in **a**. **c**, AFM image of a 2D nano-dome network with repeated 1L, 2L and 3L tri-dome units. The inset is a schematic highlighting the boundaries among domes and the layer numbers.

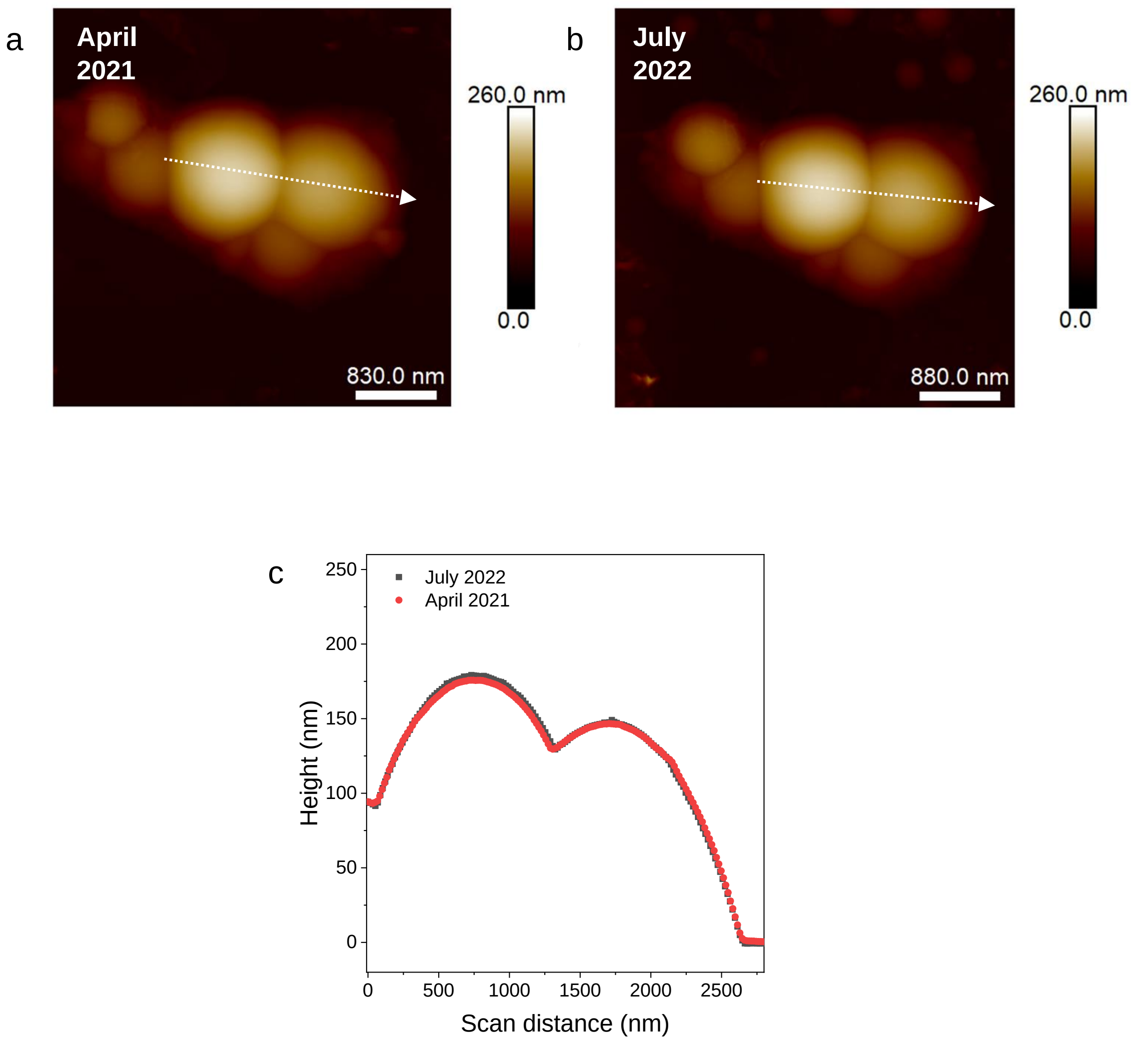


**Supplementary Figure 13 | Durability test to confirm the robustness of joint domes. a-b**, AFM images of a 1D nano-dome chain captured at two different times, April 2021 (**a**) and July 2022 (**b**) respectively. **c**, Height profiles of the same 1D dome chain along the white dashed line shown in **a**, **b**; height data extracted from the latest measurement (grey dots) well matches the one from the image obtained 15 months previously (red dots). Both samples were fabricated 3 years ago and remain nearly unchanged with time.

## Supplementary Information Note 1

## Extraction of the 2D modulus, interlayer adhesion energy, and internal pressure using inferred methods

Pressurised domes provide a stable platform to measure the 2D modulus ($E_{2D}$) of strained TMDs. By using nano-indentation and AFM, the stiffness ($k$) of the membrane and domes' profile can be measured, and the $E_{2D}$ can be computed based on these experimental values. For the $E_{2D}$ of a pressurised membrane, it can be inferred using (in the small linear regime)[1]

$$E_{2D} \approx \frac{{A_{h0}}^2 \log\left(\frac{1}{\rho_{in}}\right)}{2\pi A_\tau} \frac{R^2}{{h_m}^2} k \tag{S1}$$

where $A_{h0}$ = 0.70±0.01, $A_\tau$ = 0.40±0.08[2] (Supplementary Table 1), $k = F/\delta$ (from nano-indentation), $R$ and $h_m$ are the dome's radius and maximum height, respectively and $\rho_{in} = R_{indent}/R$ (where $R_{indent}$ is the radius of the indenter)[1].

| | $WS_2$ | $MoS_2$ | $WSe_2$ |
|---|---|---|---|
| $\upsilon$ | 0.22[3] | 0.25[3] | 0.19[3] |
| $A_{h0}$ | 0.70 | 0.69 | 0.71 |
| $A_\tau$ | 0.40 | 0.41 | 0.39 |

**Supplementary Table 1**: Poisson ratio of TMD materials and the corresponding variation $A_{h0}$ = 0.70±0.01, $A_\tau$ with $\upsilon$.

Through experiments, we found the layer dependent $E_{2D}$ of $WS_2$ is 241.5±22.2, 425.2±37.8 and 540.1±31.8 N/m for 1-3L, respectively. The same method was also applied to obtain the values for $MoS_2$ domes formed in different layers and the results are shown in Supplementary Figure 4a. In this work, the $E_{2D}$ values are slightly higher than previously reported values[4]. This is because in our work, the Poisson ratio of materials have been taken

into account in the determination of $A_h$ and $A_\tau$, which affect the final results of $E_{2D}$. Pressurised domes form due to the competition between inside pressure and interlayer vdWs energy, and this means dome samples are a good candidate to study the adhesion energy of layered materials, and the adhesion energy (γ) is given by[5,6]:

$$\gamma = \left(\frac{h_m}{R}\right)^4 \frac{5\zeta(v,q)f^2(v,q)E_{2D}}{(1-v^2)} \tag{S2}$$

$$f(v,q) = \frac{q}{8}\left(\frac{2q-1-v}{q-1}\right) \tag{S3}$$

$$\zeta(v,q) = (1+v)\frac{q-1-g(v,q)}{q} + \frac{g^2(v,q)+1+2vg(v,q)}{2(q-1)} \tag{S4}$$

$$g(v,q) = 2q-1-\left(\frac{4q(q-1)}{2q-1-v}\right) \tag{S5}$$

where $q$ is the dome profile factor where we use 2.1 obtained via statistical analysis, and $v$ is the materials' Poisson's ratio[5,6]. Using the stiffness values from layered dependent domes and Eq. (S1) to obtain the corresponding $E_{2D}$, we calculated the $\gamma$ in different layers of $WS_2$ flakes to be 33.5 ± 3.4, 42.8 ± 2.9 and 45.6 ± 6.0 meV/Å$^2$, all of which are in good agreement with the DFT results of 30.1, 40.0 and 43.0 meV/Å$^2$ for 1L-3L domes, respectively (Fig. 3b). At the same time, the experimentally obtained values for $\gamma$ of $MoS_2$ are also resolved and are summarised in Supplementary Figure 6b.

To further explore the mechanisms of micro- and nano- TMD domes, the gas pressure capped in the domes composed of different layers is required. As the amount of pressure capped in domes is mainly determined by $\gamma$ between the topmost layers and the bulk substrate, the pressure is proportional to the material's $E_{2D}$, layer number and height profile. In our case, having experimentally obtained the stiffness values of TMD domes formed in different layers and measuring the $h_m$ and $R$, Eq. (S6) below can be used to calculate the pressure of hydrogen gas stored in each dome[1]:

$$P \approx \frac{\log\left(\frac{1}{\rho_{in}}\right)}{2\pi A_\tau A_{h0}} \frac{h_m}{R^2} k \qquad \text{(S6)}$$

where all variables have been previously introduced.

Using the equation above, we found that the pressure is exponential to $1/R$ and this trend is consistent with the fact that smaller domes have larger internal pressure. As shown in Supplementary Figure 4, different layered domes have distinguished capability to pressurise different amounts of hydrogen gas when their sizes are similar. 2L and 3L domes can compress far more hydrogen molecules within the same volume, leading to a difference in pressure of at least 50 atm compared with the same sized 1L dome. This trend is confirmed by numerical computation, and the results closely match the experimental values.

**Strong boundary condition justification**

In this work, a clamped boundary condition is applied as the 2D interface condition when the nano-indentation measurements were conducted on dome samples for the mechanical features and adhesion energy of different layered TMD films. The justifications of the strong boundary condition are detailed below.

Firstly, when nano-indentation was employed to measure the stiffness, there is no obvious shape deviation induced by slippage of the TMD films during and after the load is applied. Supplementary Figure 13 shows the shape profile of two adjoining domes, sampled over a one-year period, and the shape profile is maintained over long periods of time which strongly supports a strong clamping at the edges of domes. Moreover, the indentation has little change on the volume of the dome. The AFM tip can be seen composed of a hemisphere as the top part and as a conical frustum at the bottom, so the indentation volume ($V_{\text{indentation}}$) can be estimated based on the indentation depth ($\delta$) and the AFM tip geometry with tip radius ($R_{indent}$ = 8nm) and the average apex angle ($\theta$ = 19°). For the volume of conical frustum (bottom), it can be calculated by the equation as follows:

$$V_{\text{bottom}} = \frac{\pi(\delta - R_{indent})}{3}\left[{R_{indent}}^2 + R_{bottom}(\delta)^2 + (R_{bottom}(\delta)\ + R_{indent})^2\right] \quad \text{(S7)}$$

Where $R_{bottom}(\delta) = (\delta - R_{indent})\tan(\theta) + R_{indent}$, representing the corresponding radius of total tip intruded into the dome sample with depth ($\delta$).

For the hemisphere (top) part, the volume can be described as:

$$V_{\text{top}} = \frac{2}{3}\pi\delta - {R_{indent}}^3 \quad \text{(S8)}$$

So the indentation volume ($V_{\text{indentation}}$) can be obtained by sum of two parts:

$$V_{indention}(\delta) = \ V_{\text{top}} + V_{\text{bottom}} \quad \text{(S9)}$$

Taking the dome sample shown in Fig S2 as an example. The volume ($V_{dome} = \frac{\pi}{2}R^2h$) of dome sample with $h$ = 287nm and $R$ = 1472 nm is 1.104 $\mu m^3$. On the other hand, when a 100 nN load is applied, the AFM tip with $R_{indent}$= 8 nm can only create the small amount of indentation depth, $\delta$ = 12 nm, resulting in the indentation volume $V_{\text{indentation}}$ = 2.02 $\times 10^{-6}$ $\mu m^3$. Comparing the indentation volume with dome's total volume, $V_{indention}(\delta)/V_{dome}$, the indentation only creates less than 0.0002% volume deviation, which can be considered negligible. This is indicative of our reasoning for why we deliberately used smaller radius tips to reduce the influence of the indenters effect on domes during indentation. The van der Waals adhesion energy, thus, is believed to be quite capable of preventing slippage induced effects due to such small volume deviation. Given the AFM profiling over time in conjunction with our small indenter tip, this is highly supportive of a strongly clamped boundary condition.

Secondly, our boundary assumption is consistent with previous reports[5-7]. The clamped boundary condition is a universal boundary assumption in other works and our related previous work for both proton irradiation and stacking methods. In our previous work[5], we used Raman to determine the Grunesian parameter and strain along the radial direction of domes that well

matched the theoretical predictions, and the modelling assumption was based on a strongly clamped boundary condition.

Therefore, the clamped boundary condition employed in this work is valid, and the conclusions and calculations analytically and numerically support the experimental results with quite high accuracy.

**Supplementary Information Note 2**

**Numerical Simulations using Finite Element Analysis (FEA)**

For the COMSOL numerical implementation, COMSOL 5.6 was used to numerically calculate the dome profiles, height, stress and strain distributions, and stiffness based on the experimentally obtained AFM profiles and indentation curves. Both 2D axisymmetric model and 3D modelling of different membranes and their adjoining domes are modelled using the membrane module for the height, strain and stiffness distributions for all dome calculations used throughout this work provided in the main text and in the supplemental information.

Material parameters used in the simulations were derived from the method introduced in Supplementary Note 1, therefore, for each different dome, a different 2D modulus was used for single domes and double dome simulations. Fixed boundary conditions (clamped) were used for the membrane boundary conditions. For the indentation to stiffness simulations, the experimental indentation depth at 10 nN ($F$) for each dome was used and the corresponding indentation ($\delta$) was converted to an equivalent surface area using the spherical tip indenter approximation to calculate the new equivalent indenter surface area ($A_{in}$), which results in a new equivalent radius for the indenter (i.e, indentation depth to equivalent contact surface area)[8]. The force was then converted to a contact pressure over the $A_{in}$ for indentation simulations *i.e.* $P_{in}(\delta)=F(\delta)/A_{in}(\delta)$. Moreover, a 10 nN ($F(\delta)$) was used as most domes at different locations had similar indentation depth at this range (if they had similar properties in Supplementary Note 1, otherwise, the corresponding $\delta$ is used to calculate the equivalent $A_{in}$), therefore, to maintain consistency this indentation force was used to verify stiffness values. Small deviations in indenter size have a logarithmic dependency which can cause large variations as discussed in Supplementary Note 7.

This indentation method was applied to generate Figures 2g and h in the main text. Using an axisymmetric 2D model, we applied the equivalent $A_{in}(\delta)$ from the experimental data obtained via AFM (this was consistent for domes in the low indentation regime), the two conditions of the original dome height and difference in height induced by the pressure applied over the equivalent $A_{in}(\delta)$ resulted in the generation of the numerical stiffness value curves (N/m). As noted, during numerical simulations, we only used two conditions being no indentation and the indentation from a resultant 10 nN force, and as shown in Figures 2g and h, this was sufficient for modelling the mechanical stiffness of domes which matched very well with the experimental results obtained via AFM indentation. To incorporate the effect of layer number, we also increased the thickness of our membrane model accordingly and used the derived 2D modulus values from Supplementary Note 1. This approach was quite successful for modelling the stiffness between different domes and matched very well with the experimental stiffness maps obtained.

For the position dependent indentation curves and the resultant height profiles for adjoining domes, a 3D model was used instead of a 2D axisymmetric formulation to allow for a small indenter radius at various positions along the scanning distance co-ordinate of the membrane for numerically simulating position dependent membrane indentations as shown in Figures 4d and Supplementary Figures 2d, 8d and 9d. To enable the simulations involving adjoining domes, the transition between different domes with different thickness was achieved using the identity edge pair function in COMSOL as the simplest method to simulate this approach. The identity edge pair location was determined from the experimental geometry profiles obtained *via* AFM. We simply had to use the measured geometry from AFM in terms of the domes boundaries as our COMSOL model uses finite element analysis, which only accounts for continuum mechanics and not a first principles energy calculation through molecular dynamics. This may be of interest for future work, however, the authors believe that

the computational requirements would be extensive and angle minimisation would also need to be incorporated, therefore, COMSOL was a more practical approach for determining height profiles and stiffness of adjoining domes as shown throughout the manuscript and this supplemental work. From the comparisons in Fig. 4, and Supplementary Figures 8 and 9, it appears that this boundary condition was sufficient for numerically obtaining the height and stiffness profiles of coalescing domes. The same indentation technique was applied between the 2D and 3D models for consistency which in the low regime of stiffness measurements appears to match quite well with our experimental results.

We would also like to note, we specifically incorporated the use of a small indenter for the experiments which aided numerical simulations due to the indenter radius << dome footprint radius, thus heavily minimising nonlinear effects due to large surface area changes due to indentation. Additionally, in the low indentation regime, nonlinear mechanics of the domes themselves have not been encountered in the form of polynomial stiffness curves, however, the modelling in this work may be extended to this. Whilst we use clamped boundary conditions throughout the simulations and analytical model, which matched very well with our experimental results after a long period of testing coupled with several indentation tests, realistically there is more than likely slippage and other phenomena which was not able to be simulated or not yet well understood. Moreover, there are many little side domes around the edges and there may be an in-plane vdWs adhesion or Casimir forces which appear not to have any influence on the simulations conducted in this work in verifying our domes mechanical properties and resulting variant Plateau’s law angles.

## Supplementary Information Note 3

## Dome shape vs in-plane stiffness variation

To understand the influence of various dome footprints on measured stiffness, we performed a COMSOL simulations based on possible dome shapes as shown in Supplementary Figure 3. The same amount of pressure is applied into the domes with varied geometries that deviate from a perfectly round dome such as ovals and a round-rectangular dome. The in-plane stiffness from the ideal dome with a circular footprint is 9.47 N/m, while the $k$ values that have an oval shape with 10% and 20% shape deviation in the x-y directions have $k$ values 9.27 and 8.83 N/m, respectively, indicating a 2.1% and 6.7% drop in stiffness. Moreover, the stiffness value from a round rectangular shape is also 9.27 N/m, leading to a 2.1% drop compared with the ideal dome scenario. Therefore, although the deviation of dome shape has some influence on the stiffness, the small deviation of in-plane stiffness indicates it is reasonable to use the round shape as a universal shape for the simulation and prediction of the in-plane stiffness from random domes. The circle in the centre is the indenter footprint and load location.

**Supplementary Information Note 4**

**Pressure estimation *via* different approaches**

To examine the presence of pressurised $H_2$ molecules within the domes, the first method used is to place the $H^+$ irradiated $WS_2$ dome sample in a low temperature environment (21 K) and monitor the inflation of domes with increased temperature. At low temperature (T = 21 K), $WS_2$ domes remained deflated due to the liquid form of trapped $H_2$ molecules under an elastic membrane. When the temperature is raised, the gaseous phase transition of $H_2$ molecules occurs, this leads to elastic balloon-like structures containing gas at different pressures. Therefore, the different phase transition temperature ($T^{H}_{LV}$) for dome inflation could effectively indicate the corresponding pressure level within the domes. As shown in Supplementary Figure 5a, when the environment temperature was at 27 K, no $WS_2$ domes on the flake were observed, meaning that the pressure in all domes is larger than 5.4 atm. But at 28 K, the first dome inflation occurred, labelled as dome #1, and the corresponding pressure could be estimated as 5.8 atm referring to Supplementary Figure 5b. As temperature was increased by 1 and 2 K, more domes appeared, and their pressure could be determined at 6.8 and 8 atm for $T^{H}_{LV}$ = 29 and 30 K, respectively. The pressure results obtained by the first method are summarized in Supplementary Table 2 below. Through comparing the radius of domes with different $T^{H}_{LV,}$ we found that smaller domes would appear at relatively higher temperature which corresponded to higher pressure. This trend is consistent with the trend demonstrated in Supplementary Figure 4. In our previous work[9], the statistics data demonstrated that most of domes would deflated when their phase transition temperature is $T^{H}_{LV}$ = (32.2±2.4) K, which represent the capped pressure of most domes ranges from 9 to 20 atm.

Moreover, nano-indentation as the second method was performed onto the selected domes and the stiffness values (*k*) were obtained under ambient conditions. The radius, height

and stiffness values for domes 1 to 5 were extracted. Based on the inferred method (Supplementary Note1), the pressure inside the domes were extracted to be 6.5, 7.2. 7.7, 8.2 and 8.1 atm accordingly, all of which are summarized in Supplementary Table 2 below. Comparing the pressure results governed by two different methods, they are comparable and have a good agreement, despite the small discrepancy which may arise from temperature induced modulated mechanical properties of TMD films. This indicates the inferred method employed in our work is capable to effectively probe the pressure condition in the domes.

| ***1. Phase transition method*** | | | ***2. Nano-indentation method*** | | |
|---|---|---|---|---|---|
| Dome No. | $T^{H}_{LV}$ (K) | $P_{exp1}$ (atm) | Dome No. | $R$ at $RT$ (nm) | $P_{exp2}$ (atm) |
| 1 | 28 | 5.8 | 1 | 3847 | 6.5 |
| 2 | 29 | 6.8 | 2 | 3622 | 7.2 |
| 3 | 29 | 6.8 | 3 | 3364 | 7.7 |
| 4 | 30 | 8.0 | 4 | 3472 | 8.2 |
| 5 | 30 | 8.0 | 5 | 3515 | 8.1 |

**Supplementary Table 2**: Capped gas pressure of selected domes estimated by two methods: hydrogen phase transition with temperature (blue) and nano-indentation method (green).

**Supplementary Information Note 5**

**DFT simulations involving adhesion energy of different TMD layers**

The plane-wave method in the framework of DFT as implemented in QUANTUM ESPRESSO code[10] is employed. Pseudopotentials are generated using the scheme proposed by Troullier and Martins (TM)[11]. GGA with the Perdew-Burke-Ernzerhof (PBE)[12] is used for the exchange-correlation effect. To accurately account for nonlocal the van der Waals interaction between layers, we apply the nonlocal correlation functional method of Ref[13]. The energy cutoff of 70 Ry is chosen for plane-wave function's expansion. The *k*-point samplings are 12×12×1 and 12×12×3 for monolayer and crystal unit cells, respectively. The cell parameters and atomic positions are optimized simultaneously. The binding energy, $E_b$, between layers of TMD is calculated using the approach in Ref[14]. To simulate the thermal expansion effect, the thermal expansion coefficients of single layer and bulk TMD are employed[15-17].

## Supplementary Information Note 6

## Bursting test to confirm the configuration of joint domes

To gather more direct evidence that a joint dome is made up of multiple domes within different basal planes of the TMD, a joint tri-dome with 1L, 2L and 3L dome regions (Supplementary Figure 8) was selected for a bursting test. The 1L dome in Supplementary Figure 8a was deliberately burst using AFM indentation to explore the remnant structures. AFM height profile, stiffness mapping and SHG imaging before and after the bursting of the 1L dome were shown in Supplementary Figure 8. The assignment of the 1L, 2L and 3L domes were confirmed by the stiffness mapping (Supplementary Figure 8b) and SHG imaging (Supplementary Figure 8c). As shown in Supplementary Figure 8c, the 1L dome (used for bursting test) region had a strong SHG signal, while the 2L dome had no SHG signal. After the bursting of the 1L dome, a 2L region (B region in Supplementary Figure 8e) and a 1L region (A region in Supplementary Figure 8e) coexist over the 2L dome's surface simultaneously. The stiffness of A region is significantly lower than the stiffness of B region (Supplementary Figure 8h), confirming that A region is 1L (B region is 2L). This bursting test directly confirms the configuration of a joint bi-dome, as shown in Supplementary Figure 8g.

**Supplementary Information Note 7**

**Mechanics of thin domes**

In this section, a formulation for the mechanics of thin domes is presented. Domes are characterised by their height profile which has been shown to be universal for dome and tent like structures given by[5,6]

$$h = h_m\left(1 - \left(\frac{r}{R}\right)^q\right) \tag{S10}$$

where *h* is the out of plane displacement, $h_m$ is the maximum height of the dome at the centre (as noted previously), $r$ is the radial distance along a dome, $R$ is the radius of the dome's footprint (also noted previously) and $q$ is a constant. $q$ has been shown to equal 2-2.2 for domes[5] (we use $q$ = 2.1) and 2/3 for nanotents[6]. To solve the in-plane displacement field, the in plane equilibrium equation can be solved using the Foppl-von-Karman equation in the membrane limit given by[1,5]

$$\frac{d^2u}{dr^2} + \frac{1}{r}\frac{du}{dr} - \frac{u}{r^2} = -\frac{1-v}{2r}\left(\frac{dh}{dr}\right)^2 - \frac{dh}{dr}\frac{d^2h}{dr^2} \tag{S11}$$

where *u* is the in-plane displacement. Whilst there have been several different formulations for the solution of Eq. (S11) depending on the assumptions of the out of plane displacement.[8,18] For this solution, we adopt the solution provided by Blundo et al. and Dai *et al*[5,6]. Solving out the second order differential equation for *u*, results in

$$u = \frac{f(v,q)h_m^2}{R}\left(\left(\frac{r}{R}\right) - \left(\frac{r}{R}\right)^{2q-1}\right) \tag{S12}$$

Having the radial displacement, the radial and circumferential strain fields along the radius of a dome can be formulated according to:

$$\epsilon_{rr} = \frac{du}{dr} + \frac{1}{2}\left(\frac{dh}{dr}\right)^2 \tag{S13}$$

$$\epsilon_{\phi\phi} = \frac{u}{r} \tag{S14}$$

Accordingly, the radial and circumferential strain fields in the strong shear region are formulated as[5,6]

$$\epsilon_{rr} = \frac{f(v,q)h_m^2}{R^2}\left(1 - \frac{1+v-2qv}{2q-1-v}\left(\frac{r}{R}\right)^{2q-2}\right) \tag{S15}$$

$$\epsilon_{\phi\phi} = \frac{f(v,q)h_m^2}{R^2}\left(1 - \left(\frac{r}{R}\right)^{2q-2}\right) \tag{S16}$$

From the formulation of the strain field, the corresponding radial and circumferential stresses along the radius of a dome can be formulated as

$$N_{rr} = \frac{E_{2D}}{1-v^2}\left(\epsilon_{rr} + v\epsilon_{\phi\phi}\right) \tag{S17}$$

$$N_{\phi\phi} = \frac{E_{2D}}{1-v^2}\left(\epsilon_{\phi\phi} + v\epsilon_{rr}\right) \tag{S18}$$

With the above definitions, a set of coupled nonlinear differential equations can be formulated for the solution of the transcendental equations given by Eq. (S11) and Eq. (S12)

$$D\left(\frac{d^3h}{dr^3} + \frac{1}{r}\frac{d^2h}{dr^2} - \frac{1}{r^2}\frac{dh}{dr}\right) - \frac{dh}{dr}N_{rr} = \frac{r}{2}\Delta p \tag{S19}$$

where $D$ represents the neglectable bending stiffness of the dome and $\Delta p$ represents the internal pressure difference inside the dome. $D$ is given by[5,6,19]

$$D = \frac{E_{2D} t^2}{12(1 - \nu^2)} \tag{S20}$$

where $t$ is the thickness.

In this work, the authors have used COMSOL Multiphysics 5.6 to solve the corresponding large deformation membrane equations for displacement profiles, stress, strain, and stiffness (stiffness method mentioned in Supplementary Note 2). Now knowing all the terms above, an energy formulation for the mechanics of domes can be formulated as[5,6,8]

$$U_{total} = U_{bending} + U_{stretching} + U_{vdW} + U_{pressure} \tag{S21}$$

By minimising $U_{total}$ with respect to $h$ and $r$, the solutions for the internal pressure and vdWs adhesion energy can be derived ($U_{bending}$ is normally ignored in the analysis due to the very small influence it has). The resulting analytical formulation of the adhesion energy is given in Eq. (S2) presented in the previous section.

**Indentation of pressurised domes**

In the following section, a discussion of the effects of indentation of the domes which are highly pressurised thin membranes is discussed. By realising that the effect of a finite radius indenter compared to the radius of the dome results in a logarithmic term[1]. The elastic energy resulting from a finite indenter results in[8]

$$E_{elastic} = T_{eff} \int_0^{R^*} 2\pi r dr \left(\frac{dh}{dr}\right)^2 \tag{S22}$$

where $T_{eff}$ is the effective tension of the membrane. Vella and Davidovitch[1] provided a comprehensive theoretical formulation for the indentation metrology of clamped ultrathin

sheets for a variety of different indentation sizes and indentation depths between the small and large indentation schemes, resulting in linear and nonlinear behaviours. From the formulations, in the small indentation regime[1]

$$F = \frac{2\pi T_{eff}\delta}{\ln\left(\frac{1}{\rho_{in}}\right)} \tag{S23}$$

where $F$ is the indentation force, $\delta$ is the indentation depth and $\rho_{in}$ is the ratio of the radius of the indenter to the radius of the dome. Eq. (S23) can be rearranged into the following form[1]

$$\frac{F}{\delta} = \frac{2\pi T_{eff}}{\ln\left(\frac{1}{\rho_{in}}\right)} \tag{S24}$$

The left-hand term in Eq. (S24) is the stiffness of the dome which can be experimentally determined using AFM having known the dome geometry and the indenter radius from AFM calibration. It is convenient to define the effective tension of a membrane along the radial co-ordinate as[1]

$$T_{eff} = \frac{N_{rr}(r) + N_{\phi\phi}(r)}{2} \tag{S25}$$

The effective tension along the radius of a dome using the $r$ component is shown in Supplementary Figure 2d, and the effective tension at the centre of a dome is governed when $r = 0$.

The above formulation for the mechanics of thin domes and their indentation reveals interesting phenomena which needs to be explained to completely understand the system. From the dome geometry, the strain profile of domes is independent of material and layer number, as the strain is a quantity describing deformation induced by an applied load. The stress

functions in the radial and circumferential directions are a function of the 2D modulus of the material. In this work, we fabricated 1L, 2L and 3L domes and it is expected that the 2D modulus should increase with layer number and this has been shown for suspended materials subjected to AFM indentation[20]. It is expected that $E_{2D}$ is larger for multiple layer domes resulting in increased pressure to achieve the same height and an increased stiffness (see Eqs. (S17), (S18), (S24) and (S25)). Moreover, for larger $E_{2D}$ in 2L and 3L systems, it is expected that that corresponding $\gamma$ also increases due to the exfoliation energy required to separate multiple layers from the initial few basal plane layers.

**Supplemental Information Note 8**

**Plateau's law in liquid bubbles and variations in joint angle in analogous nano dome networks**

Energy minimisation problems exist everywhere in the universe from large scale systems, macro, micro and in nanoscale systems. Such energy minimisation topologies are abundant in nature in the formation of cell structures and bees forming honeycombs. Probably, the most famous energy minimisation problem is the formation of liquid soap bubbles and the interaction of merging bubbles to assume a minimum energy formation. As bubbles merge, smaller bubbles with larger pressure always protrude into larger bubbles. Similar to the hexagonal structure of graphene, liquid soap bubbles form at 120° angles to assume a minimum energy shape and this is considered the double bubble conjecture[21]. However, if soapy liquid merges with four vertices then they form the Maraldi angle at 109.5°. It is very easy to observe that liquid bubbles always rearrange themselves for a minimum energy state in terms of their surface area to volume ratio. From Laplace's law, we have the pressure inside a half sphere is given by:

$$\Delta p = \frac{2\sigma}{R_s} \tag{S26}$$

where $\Delta p$ is the pressure differential between inner and outer wall of a bubble (same terminology for internal pressure for a dome), $\sigma$ is the surface tension at the liquid surface interface (N/m) and $R_s$ is the Radius of the sphere.

$\sigma$ is a measure of the cohesive forces in a liquid or biological membrane. Therefore, the pressure difference in liquid bubbles is always inversely proportional to their radius and the surface tension of the liquid which is a constant for a given set of thermodynamic parameters (i.e. changes with pressure and temperature of the surrounding environment). For our case, we assume standard operating conditions of room temperature and ambient pressure. For ambient

conditions, the surface tension of soapy liquid bubbles is 25 mN/m[22]. From Plateau's law, we know that soap bubbles always meet at 120° angles to assume a minimum energy shape, therefore the force equilibrium of soap bubble configurations results in[23]

$$\sigma_{ij} = \sigma_{ki} \cos(\pi - \phi_i) + \sigma_{jk} \cos(\pi - \phi_j) \tag{S27}$$

$$\sigma_{ki} \sin(\pi - \phi_i) = \sigma_{jk} \sin(\pi - \phi_j) \tag{S28}$$

where $i$, $j$ and $k$ denote three arbitrary boundaries. From Eqs. (S27) and (S28), it can be rearranged for the angle between two adjacent bubbles as

$$\phi_i = \arccos\left((\sigma_{jk}^2 - \sigma_{ij}^2 - \sigma_{ki}^2)/(2\sigma_{ij}\sigma_{ki})\right) \tag{S29}$$

From Eq. (S29), it is easy to note that for the case of liquid soap bubbles that all the surface tension components are equal and Eq (S29) reduces to

$$\phi_i = \arccos\left(-\frac{1}{2}\right) = 120° \tag{S30}$$

Eq. (S30) demonstrates Plateau's law for angle minimisation in soapy liquid bubbles. However, small deviations from this angle have been reported in foams.[24] These deviations can be attributed to the thickness dependence of the surface tension and this was shown using a disjoining isotherm[23]. The thickness reduction effect on the surface tension provides an ideal platform for investigations involving 2D materials. 2D materials have highly sensitive parameters dependent upon the thickness of the layer and the corresponding $E_{2D}$ as shown in previous sections of this Supplementary Information. After the total formation of the energy of a single nano dome given in Eq. (S21), the pressure difference can be formulated as[5,6]:

$$\Delta p = \frac{q+2}{q}\left(\frac{4\zeta(v,q)E_{2D}}{1-v^2} f^2(v,q)\left(\frac{h_m}{R}\right)^3 + 2\mu(v,q)D\left(\frac{h_m}{R^3}\right)\right)\frac{1}{R} \tag{S31}$$

where all previous terms are the same as previously introduced in Note 1 (Eqs. (S2-S5)) and

$$\mu = q^2\left(\frac{q + 2(q-1)v}{2q-2}\right) \tag{S32}$$

After rigorous searches and statistics of nano domes in the literature, $q$ was found to be within 2.0-2.2[5], which is consistent with the our current work and a value of 2.1±0.05 was extracted using Eq. (S10)

Eq. (S31) can be rearranged in a similar format analogous to Laplace's law for liquid bubbles given by

$$\Delta p = \frac{2\sigma}{R} \tag{S33}$$

the $\sigma$ is given by

$$\sigma = \frac{1}{2}\left[\frac{q+2}{q}\left(\frac{4\zeta(v,q)E_{2D}}{1-v^2}f^2(v,q)\left(\frac{h_m}{R}\right)^3 + 2\zeta_2(v,q)D\left(\frac{h_m}{R^3}\right)\right)\right] \tag{S34}$$

However, since our domes have negligible bending stiffness, the effective surface tension is given by

$$\sigma = \frac{q+2}{2q}\left(\frac{4\zeta_1(v,q)E_{2D}}{1-v^2}f^2(v,q)\left(\frac{h_m}{R}\right)^3\right) \tag{S35}$$

In this work, an analytical expression for $\sigma$ of TMD nano domes is formulated which includes geometrical functions, the universal $h_m/R$ ratio and the only parameter that changes due to thickness dependence is the $E_{2D}$ which was shown in the stiffness mapping and FEA in previous Supplementary Notes.

Moreover, to have better understanding of variations from Plateau's law in our dome network systems, it is worthy to analyse the force equilibrium in our case and the reason why the variations exist in joint angles. According to Eqs. (S34) and (S35), the effective surface

tension of a dome is determined by the inner pressure of domes and is proportional to the $E_{2D}$, thus the difference in effective surface tension between the layers of a joint dome would cause joint angles larger/smaller than 120°. Referring to the previous section, in our double dome case, the surface tensions can be classified into $\sigma_1$, $\sigma_2$ and $\sigma_1'$, where $\sigma_1$, and $\sigma_2$ represent the effective surface tensions of a normal 1L dome and 2L dome sitting on a bulk TMD substrate, respectively, but $\sigma_1'$ means the projected component on the TMD substrate plane (in the same plane with $\sigma_1$ and $\sigma_2$) effective surface tension from the shared boundary between the 1L dome and 2L dome as in Supplementary Figure 10a.

Due to the configuration of the 2L region of the double dome, it is composed by two monolayers. The top single layer is continuous with the adjacent monolayer dome, and the bottom monolayer forms the shared wall to separate hydrogen gas between two domes. So, the surface tension from the bilayer region of the joint dome is only generated by the bottom single layer and relevant adhesion energy to prevent layer sliding. Therefore, the effective surface tension is the difference in the surface tensions of the 2L film and 1L film given by

$$\sigma_2^{eff} = \sigma_2 - \sigma_1 \tag{S36}$$

For the surface tension of the bottom single layer on the shared boundary, the exfoliation process should mean that $\sigma_1'$ represents a portion of $\sigma_1$ as it should require less energy to detach a 1L sheet from a 2L sheet rather than from the bulk. As the shared boundary can be regarded as the exfoliation of a top single layer from a bilayer film, we propose the adhesion energy for this process is calculated as:

$$\gamma^{L_{12}} = 2 \times \gamma^{1L} - \gamma^{2L} \tag{S37}$$

where $\gamma^{1L}$ and $\gamma^{2L}$ are the adhesion energy of 1L and 2L domes, respectively. Similarly, the surface tension of the shared boundary can be calculated as

$$\sigma_1' = \sigma_1(\gamma^{L_{12}}/\gamma^{1L}) \quad \text{(S38)}$$

Through analysing the conditions of force components exerted at the joint point, joint angle $\alpha$ can be analytically determined. Combining Eqs. (S29 and S35-S38), we formulate[23]

$$\left.\begin{aligned} \alpha_3 &= arccos\left[\frac{(\sigma_1')^2 - \sigma_1^2 - (\sigma_2^{eff})^2}{2\sigma_1^2(\sigma_2^{eff})^2}\right] \\ \alpha_2 &= arccos\left[\frac{\sigma_1^2 - (\sigma_2^{eff})^2 - (\sigma_1')^2}{2(\sigma_1')^2(\sigma_2^{eff})^2}\right] \\ \alpha_1 &= arccos\left[\frac{(\sigma_2^{eff})^2 - (\sigma_1')^2 - \sigma_1^2}{2(\sigma_1')^2\sigma_1^2}\right] \end{aligned}\right\} \quad \text{(S39)}$$

Using Eq. (S39), we found that the analytical joint angles in double bubbles have great agreement with the experimental results shown in Fig. 5a in the main text and Supplementary Figure 11a. We did not extend this to the case of 3L domes and 3 adjoining domes for $\beta$ in the main text as there may be other relevant quantum effects as the number of basal planes increase. It should also be noted that for the 3L dome in a situation of 3 merging bubbles appears to be returning to the normal scenario, therefore, future work with rigorous searching conditions and if domes in deeper layers can be generated should return to Plateau's law with joint angles of 120°.

## Supplementary References


1 Vella, D. & Davidovitch, B. Indentation metrology of clamped, ultra-thin elastic sheets. *Soft Matter* **13**, 2264-2278 (2017).

2 Jensen, H. M. J. E. F. M. The blister test for interface toughness measurement. **40**, 475-486 (1991).

3 Kang, J., Tongay, S., Zhou, J., Li, J. & Wu, J. Band offsets and heterostructures of two-dimensional semiconductors. *Applied Physics Letters* **102** (2013).

4 Liu, K. *et al.* Elastic Properties of Chemical-Vapor-Deposited Monolayer $MoS_2$, $WS_2$, and Their Bilayer Heterostructures. *Nano Letters* **14**, 5097-5103 (2014).

5 Blundo, E., Yildirim, T., Pettinari, G. & Polimeni, A. Experimental Adhesion Energy in van der Waals Crystals and Heterostructures from Atomically Thin Bubbles. *Physical Review Letters* **127**, 046101 (2021).

6 Dai, Z. *et al.* Interface-governed deformation of nanobubbles and nanotents formed by two-dimensional materials. *Physical Review Letters* **121**, 266101 (2018).

7 Koenig, S. P., Boddeti, N. G., Dunn, M. L. & Bunch, J. S. Ultrastrong adhesion of graphene membranes. *Nature Nanotechnology* **6**, 543 (2011).

8 Khestanova, E., Guinea, F., Fumagalli, L., Geim, A. & Grigorieva, I. Universal shape and pressure inside bubbles appearing in van der Waals heterostructures. *Nature communications* **7**, 12587 (2016).

9 Tedeschi, D. *et al.* Controlled Micro/Nanodome Formation in Proton-Irradiated Bulk Transition-Metal Dichalcogenides. *Advanced Materials* **31**, 1903795 (2019).

10 Giannozzi, P. *et al.* QUANTUM ESPRESSO: a modular and open-source software project for quantum simulations of materials. *Journal of Physics: Condensed Matter* **21**, 395502 (2009).

11 Troullier, N. & Martins, J. L. Efficient pseudopotentials for plane-wave calculations. *Physical Review B* **43**, 1993-2006 (1991).

12 Perdew, J. P., Burke, K. & Ernzerhof, M. Generalized Gradient Approximation Made Simple. *Physical Review Letters* **77**, 3865-3868 (1996).

13 Sabatini, R., Gorni, T. & de Gironcoli, S. Nonlocal van der Waals density functional made simple and efficient. *Physical Review B* **87**, 041108 (2013).

14 Björkman, T., Gulans, A., Krasheninnikov, A. V. & Nieminen, R. M. van der Waals Bonding in Layered Compounds from Advanced Density-Functional First-Principles Calculations. *Physical Review Letters* **108**, 235502 (2012).

15 Hu, X. *et al.* Mapping thermal expansion coefficients in freestanding 2D materials at the nanometer scale. *Physical Review Letters* **120**, 055902 (2018).

16 Çakır, D., Peeters, F. M. & Sevik, C. Mechanical and thermal properties of h-$MX_2$ (M= Cr, Mo, W; X= O, S, Se, Te) monolayers: A comparative study. *Applied Physics Letters* **104**, 203110 (2014).

17 Huang, L. F., Gong, P. L. & Zeng, Z. Correlation between structure, phonon spectra, thermal expansion, and thermomechanics of single-layer $MoS_2$. *Physical Review B* **90**, 045409 (2014).

18 Yue, K., Gao, W., Huang, R. & Liechti, K. M. Analytical methods for the mechanics of graphene bubbles. *J. Appl. Phys.* **112**, 083512 (2012).

19 Jiang, J. W., Qi, Z., Park, H. S. & Rabczuk, T. Elastic bending modulus of single-layer molybdenum disulfide ($MoS_2$): finite thickness effect. *Nanotechnology* **24**, 435705 (2013).

20 Zhang, R., Koutsos, V. & Cheung, R. Elastic properties of suspended multilayer WSe2. *Appl. Phys. Lett.* **108**, 042104 (2016).

21 Hutchings, M., Morgan, F., Ritoré, M. & Ros, A. Proof of the double bubble conjecture. *Annals of Mathematics*, 459-489 (2002).

22 Román, F. L., Faro, J. & Velasco, S. A simple experiment for measuring the surface tension of soap solutions. *American Journal of Physics* **69**, 920-921 (2001).

23 Neimark, A. V. & Vignes-Adler, M. Variations from the Plateau law in foams. *Physical Review E* **51**, 788 (1995).

24 Stavans, J. & Glazier, J. A. J. P. r. l. Soap froth revisited: dynamic scaling in the two-dimensional froth. **62**, 1318 (1989).
